# Comparative and parallel genome-wide association studies for metabolic and agronomic traits in cereals

**DOI:** 10.1038/ncomms12767

**Published:** 2016-10-04

**Authors:** Wei Chen, Wensheng Wang, Meng Peng, Liang Gong, Yanqiang Gao, Jian Wan, Shouchuang Wang, Lei Shi, Bin Zhou, Zongmei Li, Xiaoxi Peng, Chenkun Yang, Lianghuan Qu, Xianqing Liu, Jie Luo

**Affiliations:** 1 National Key Laboratory of Crop Genetic Improvement and National Center of Plant Gene Research (Wuhan), Huazhong Agricultural University, Wuhan 430070, China; 2 College of Life Science and Technology, Huazhong Agricultural University, Wuhan 430070, China

## Abstract

The plant metabolome is characterized by extensive diversity and is often regarded as a bridge between genome and phenome. Here we report metabolic and phenotypic genome-wide studies (mGWAS and pGWAS) in rice grain that, in addition to previous metabolic GWAS in rice leaf and maize kernel, show both distinct and overlapping aspects of genetic control of metabolism within and between species. We identify new candidate genes potentially influencing important metabolic and/or morphological traits. We show that the differential genetic architecture of rice metabolism between different tissues is in part determined by tissue specific expression. Using parallel mGWAS and pGWAS we identify new candidate genes potentially responsible for variation in traits such as grain colour and size, and provide evidence of metabotype-phenotype linkage. Our study demonstrates a powerful strategy for interactive functional genomics and metabolomics in plants, especially the cloning of minor QTLs for complex phenotypic traits.

## Abstract (web summary)

The plant metabolome is often considered as a bridge between genomic and phenotypic variation. Here, the authors perform metabolic and phenotypic genome-wide association studies in rice grain, and compare to prior studies on rice leaf and maize kernel, to demonstrate a new strategy to investigate complex traits.

## Graphical abstract

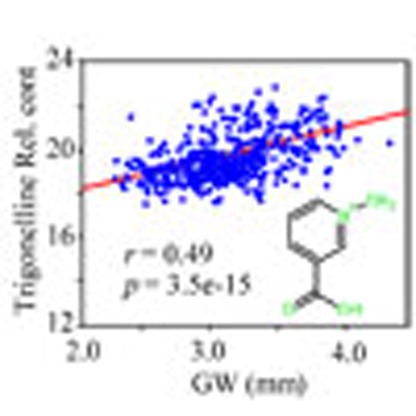

Sessile in nature, plants produce a large array of metabolites for their growth, development and adaptation to the ever-changing environment^1^,^2^,^3^. There has been increasing interest over the past decade in integrating metabolic and genetic approaches to unravel metabolic diversity and its underlying genetic variation in plants, including major crops^4^,^5^,^6^,^7^. Qualitative and quantitative variation both between and within plant species had been uncovered by advances in plant metabolomics and large scale profiling^8^. Mapping approaches have linked this variation to genetic factors^9^,^10^,^11^. These approaches have typically been performed by using linkage mapping with bi-parental populations^5^,^6^ and more recently by integrating high-resolution maps generated by using next-generation sequencing with widely targeted metabolomics^12^,^13^,^14^. Additionally, comprehensive metabolic profiling followed by association mapping using a wide collection of diverse natural or artificial mapping panels in plants has facilitated large-scale gene identification and revealed the genetic and biochemical foundations underlying plant metabolism^9^,^10^,^15^,^16^,^17^,^18^,^19^. In addition to species-level diversity, many studies have revealed that plant metabolites also accumulate in a spatio-temporal manner^17^,^20^ (especially secondary metabolites and to a lesser extent metabolites^13^,^21^). However, the mechanisms underlying the genetic control of the alterations among species and in different tissues within a species remain largely unknown^14^,^22^.

Rice and maize are the two most important crops supporting the majority of the population worldwide. These crops have been intensively studied to identify numerous metabolic and phenotypic traits^7^,^9^,^11^,^13^,^23^,^24^. In these selfing and outcrossing species, respectively, genetic analyses such as genome-wide association studies (GWAS) have shown trade-offs in power and resolution^25^. However, their closely conserved genomes suggest some shared genetic control^25^,^26^. Therefore, the combined use of the similarities and differences between these two species may provide insights into complex biological systems in both^27^,^28^.

Dissecting morphological traits has been a goal of plant scientists for a long time. Numerous loci have been detected by using both linkage and association mapping^25^,^29^,^30^,^31^,^32^. A number of genes have been cloned primarily by using linkage mapping, although the underlying mechanism has remained elusive in most cases^33^,^34^,^35^. Metabolites are regarded as a bridge between the genome and the phenome and can, in some cases, be either causes or markers of morphological traits^36^,^37^. Combined analysis of quantitative genetics (largely by quantitative trait locus (QTL) analysis) and metabolomics has helped researchers to infer genetic links between metabolic and phenotypic variation in plants. These combined studies have provided important information on the metabolic markers associated with agronomic traits^6^,^9^,^33^,^34^. Additionally, GWAS has been used to link metabolic traits with disease phenotypes and to provide insight into the regulation of chronic disorders^37^. These studies have provided the foundation for evaluating the genetic control of these two sets of traits at a higher resolution via GWAS or QTL mapping in major crops^9^,^12^,^24^,^33^,^38^,^39^.

We have previously reported a metabolic GWAS in maize kernels^15^ and rice leaves^16^. Here we report genetic analyses assisted by comparative and parallel GWAS in rice grains. Our findings resulted in the identification or annotation of both metabolites and candidate genes responsible for such metabolic and phenotypic traits as grain width, which is an important complex phenotypic and quality traits in rice.

## Results

### Metabolic profiling of rice grains

To assess the extent of the natural variation in metabolism in rice grains, we collected grain samples from a diverse global collection of 502 rice (*Oryza sativa*) accessions (Supplementary Data 1) and performed high-throughput quantification of their metabolites using scheduled multiple reaction monitoring in positive mode as a widely targeted metabolomics analysis to obtain the relative metabolite content (Supplementary Note 1). Of the 837 metabolic features detected in rice grains, 80 were identified by using authentic standards, and 230 were putatively annotated (Supplementary Note 1 and Supplementary Figs 1 and 2); these metabolites included flavonoids, amino acids and their derivatives, fatty acids, nucleic acids and their derivatives (Supplementary Data 2). To statistically assess the broad-sense heritability (H^2^) of the various metabolic traits, we conducted an analysis of variance by first considering the variations between the 2012 and 2013 harvests to be phenotypic variance derived from environmental factors (see Methods). Among the 837 metabolic features determined in the diverse global collection of *O. sativa* accessions (Supplementary Data 3), 587 metabolites were significant (*P*<0.05, two-way analysis of variance, *n*=4) on the basis of their genetic contribution. Subsequently, we performed studies on these 587 metabolites (Supplementary Data 4).

More than 90% of these metabolites had observed coefficients of variation >50% (Supplementary Fig. 3a). Upon examining individual groups of metabolites, we found that the 12 proteinogenic amino acids showed an average coefficient of variation of 77% with a range from 58% for valine to 106% for tryptamine. The flavonoids showed much higher and more varied coefficients of variation, with an average coefficient of variation of 253% and a range from 71 to 1,165% (Supplementary Data 5). The relationships among the metabolic trait contents were evaluated using Spearman's rank correlation. The levels of chemically related metabolites are often correlated. We identified high-positive correlations among metabolites including amino acids and their derivatives, nucleotides and their derivatives, flavonoids (Supplementary Data 6). Apigenin and chrysoeriol, which share common substrates and enzymes in their biosynthetic pathways, displayed a high positive association (*r*=0.84, *P*=8.52E-53, Pearson's correlation coefficient); a similar result was also obtained between the valine and phenylalanine levels (*r*=0.67, *P*=1.26E-36, Pearson's correlation coefficient). Some of the strongest negative correlations were detected between *O*-methylapigenin *C*-hexoside and 1-methylnicotinamide (*r*=-0.44, *P*=2.49E-25, Pearson's correlation coefficient) and between sinapoylcholine and 1-decanoyl-2-hydroxy-sn-glycero-3-phosphocholine (*r*=−0.54, *P*=2.21E-39, Pearson's correlation coefficient), possibly because of competition for the available methyl and choline groups, respectively (Supplementary Data 6). These findings indicated that common genetic factors controlled the contents of these metabolites. We also observed a strong correlation between metabolites from different categories, thus revealing previously unknown relationships between metabolites. Metabolites with high correlations were observed among amino acids and nucleotides in both the *indica* and *japonica* subgroups. When the relative metabolite levels were compared between the two rice subgroups, we found that on average, the *indica* subgroup accumulated higher levels of most of the flavonoids, especially *C*-glycosylated and malonylated flavonoids, in the grain, thus confirming the results obtained in leaves^16^. However, the relative levels of most amino acids tended to be higher in the *japonica* than in the *indica* subgroup, and this result also held true for most nucleic acids and their derivatives (Supplementary Data 7).

### Genetic basis of metabolism in rice grains

The distributions of broad-sense heritability (H^2^) across all metabolites revealed the extent of genetic contributions in determining the content of these metabolites. In our results, 200 metabolites displayed high H^2^ values, >0.4 (ref. 12; Supplementary Fig. 3b). In addition to the overall high H^2^ detected for secondary metabolites such as flavonoids, we also observed relatively high H^2^ values for some primary metabolites (Supplementary Data 5).

To dissect the genetic basis underlying the natural variation of metabolism in rice grains, GWAS was performed using a diverse global collection of 502 *O. sativa* accessions that were previously genotyped using the Illumina HiSeq 2000 system^40^. Metabolic-GWAS (mGWAS) was performed for both the full population (the 502 lines from the sequencing panel) and each of the two subgroups of rice, the *indica* subgroup (274 lines) and the *japonica* subgroup (151 lines), by using a linear mixed model (LMM), as previously reported^16^ (see Methods). We detected 1,489 lead single-nucleotide polymorphisms (SNPs; Supplementary Data 8) corresponding to 476 loci in at least one of the populations (Supplementary Data 9), within which 364 lead SNPs (corresponding to 408 loci) were repeatedly detected (for example, in at least two populations). A total of 56.4% of the detected metabolites (331 out of 587) had at least one significant association, with an average of 4.5 associations per metabolite. These loci showed effects of up to 53.0%, with a median of 9.0% (Supplementary Data 10). The full lists of significant and suggestive associations are presented in Supplementary Data 8 and 11, respectively, and may be used for further validation and follow-up study.

**Figure 1:**
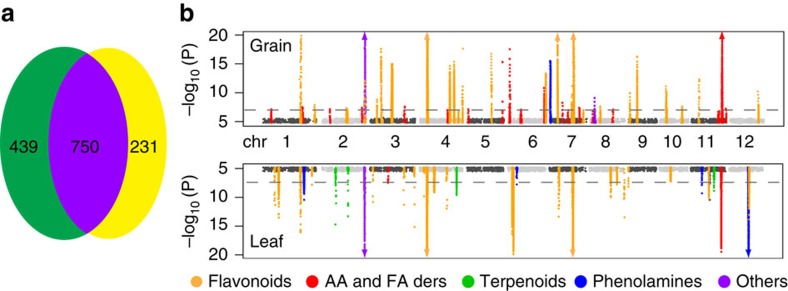
Comparison of the genetic bases of metabolism between rice grains and leaves. (**a**) Comparison of metabolic features in rice grains and leaves. (**b**) Manhattan plots of mGWAS results with genetic association in different tissues for the same metabolic features in rice. The strength of association for the grain (top) and leaf (bottom) metabolic features is indicated as the negative logarithm of the *P* value for the LMM model. All metabolite-SNP associations with *P* values below 6.6E-08 (horizontal dashed lines in all Manhattan plots) are plotted against the genome location in intervals of 1 Mb. Triangles: metabolite-SNP associations with *P* values below 1.0E-20. AA and NA ders: amino acid and nucleic acid derivatives, respectively.

The natural variation in the spatio-temporal accumulation of various metabolites has been investigated in plants^13^,^17^,^21^,^41^. When comparing the natural variation in the overall relative accumulation of metabolites in different rice tissues, we observed that the metabolism as a whole showed substantial variation between the two tissues tested. Among the 1,420 metabolic features detected in the two tissues, 439 and 231 metabolites showed specific or preferential accumulation, respectively, in only one of the tissues (Fig. 1a and Supplementary Data 12). Generally, polyphenols showed higher accumulation in the leaves than in the grains, with exception of polyphenols such as eriodictyol *O*-malonylhexoside, catechin, di-catechin, caffeic acid and peonidin *O*-hexoside (Supplementary Data 12). In contrast, some fatty acids, vitamins and their derivatives accumulated at much higher levels in the grains than in the leaves. Amino acids displayed varying accumulation patterns: the majority (including methionine, tryptamine, phenylalanine and tyrosine) accumulated preferentially in the leaves, but higher asparagine and arginine levels were detected in the grains (Supplementary Data 12).

In addition to the preferential accumulation of metabolites, we also observed the tissue-specific genetic control of metabolism. Of the 2,947 and 1,489 significant associations detected in the leaves and grains, respectively, only 105 were repeatedly detected in both tissues (Supplementary Data 13). Genome-wide analysis of the significant loci revealed 48 potential mGWAS ‘hot spots' in the grains, primarily located on chromosomes 6, 7, 9 and 11, which differed from the ‘hot spots' detected in the leaves in both number and location (52 ‘hot spots' primarily located on chromosomes 2, 6, 7 and 12) (Supplementary Fig. 4). Comparison of the loci underlying individual metabolite groups revealed that the majority of the metabolites were under different genetic control in different tissues, as reflected by both the associated loci and their effect sizes (Fig. 1b). Despite the overall distinct regulation, metabolites with moderately similar or the same genetic architecture were also detected in both tissues (Fig. 1b and Supplementary Fig. 5).

### Novel variants underlying metabolic traits in rice grains

Next, we searched for candidate genes responsible for the variation in metabolic traits in rice grains, using the combined biological and bioinformatics approaches (Supplementary Note 2). More than 30 candidate genes were newly identified, in addition to 28 previously identified genes (Supplementary Data 14). The associated SNPs were assigned by examining the mGWAS data from rice grains (Table 1).

We obtained eight candidate genes involved in the biosynthesis and transportation of amino acids and their derivatives (Supplementary Data 14). Three candidates were assigned to the levels of choline and its lysophosphatidyl derivatives. Clear signals for trigonelline, a bioactive compound that has been implicated in cell cycle control^42^, resulted in the assignment of seven candidate genes for this metabolite (Supplementary Data 14). Furthermore, our mGWAS in rice grains revealed 40 candidates (both regulatory and structural genes) involved in the biosynthesis, modification and transportation of phenylpropanoids, including the *C*-glycosyl flavones, the major class of flavonoids in cereals (Supplementary Data 14). Despite the widely reported physiological and eco-chemical functions of the *C*-glycosyl flavones, their structures and the genes responsible for their biosynthesis have been poorly investigated in rice^11^,^43^. The significant association between SNP sf0406521998 near *Os04g11970* (encoding a putative *O*-methyltransferase) and *O*-methylapigenin *C*-hexoside (*P*=6.7E-47, LMM, *n*=502) suggested that *Os04g11970* encodes an *O*-methyltransferase for this metabolite. The biochemical function of this gene was subsequently confirmed by an *in vitro* enzyme assay using the recombinant Os04g11970 protein from *Escherichia coli* (Supplementary Fig. 6). Examination of the expression patterns of the cloned and newly assigned candidate genes detected only in rice grains revealed that the majority showed exclusive or preferential expression in grains (Supplementary Data 14). This result suggested that the distinct genetic control of natural variation in metabolism in different tissues was partially determined by allelic variations in genes specifically or preferentially expressed in a tissue.

**Figure 2:**
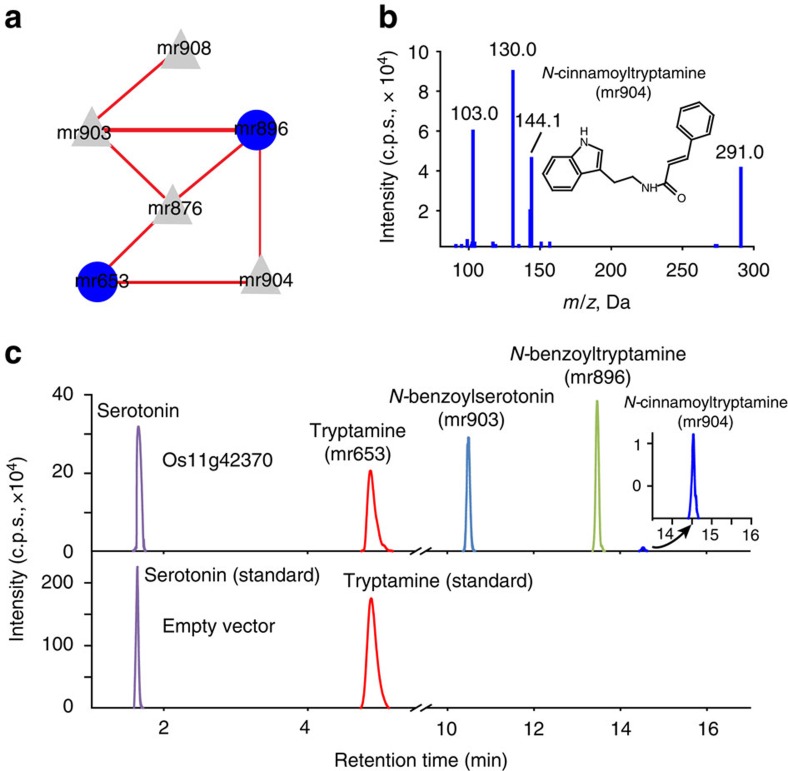
Interactive metabolite and gene identification. (**a**) Sub-network of GGM results. Blue circles: amino acid derivatives. Grey triangles: previously unknown metabolites are newly identified by GGM. The fragmentation pattern (**b**) and retention time (**c**) of *N*-benzoyltryptamine*, N*-cinnamoyltryptamine and *N*-benzoylserotonin, obtained by *in vitro* enzyme reactions catalysed by Os11g42370.

To explore the interactive metabolite and gene identifications in rice grains, we performed principal component analysis and used the Gaussian graphical model (GGM) to construct networks of directly related metabolites (Supplementary Fig. 7 and Supplementary Note 1). We observed a sub-network comprised of tryptamine, *N*-benzoyltryptamine and the unknown features mr876, mr903, mr904 and mr908; this result suggested that these metabolites were tryptamine derivatives (Fig. 2a). This possibility was supported by the fact that mr876 and mr904 showed a major m/z 144 fragment, which is the main ion for tryptamine (Supplementary Fig. 8a), whereas mr903 and mr908 showed the m/z 160 fragment, which is the typical ion for serotonin (5-hydroxyl tryptamine), in their structures (Supplementary Fig. 8b). Interestingly, the levels of three of the unknown metabolites (mr903, mr904 and mr908) and *N*-benzoyltryptamine (mr896) were strongly associated with SNP sf1125034484 in proximity to *Os11g42370*, which encodes a putative transferase (Supplementary Fig. 9 and Supplementary Data 8), thus suggesting that these features were acylated derivatives of tryptamine or serotonin. The metabolites mr876, mr904, mr903 and mr908 were tentatively assigned as *N*-acetyltryptamine, *N*-cinnamoyltryptamine, *N*-benzoylserotonin and *N*-salicyloylserotonin, respectively, on the basis of examination of their fragmentation spectra and exact masses (Supplementary Fig. 8 and Supplementary Data 15). These assignments were confirmed by comparing their retention times and fragmentation patterns with the products of recombinant Os11g42370 (Fig. 2b,c). The putative annotation of these phenolamides strongly suggested that *Os11g42370* encodes a novel BAHD (Benzoyl alcohol O-acetyltransferase, Anthocyanin O-hydroxycinnamoyl transferase, Anthranilate N-hydroxycinnamoyl/benzoyl transferase, Deacetyl vindoline 4-O-acetyltransferase) acyltransferase that catalyses the biosynthesis of the tryptamine/serotonin-derived phenolamides reportedly catalysed by the GCN5-related N-acetyltransferases (GNAT) enzymes^44^. These findings provide new insights into the biosynthesis of these stress-responsive phenylpropanoids in rice grains.

**Figure 3:**
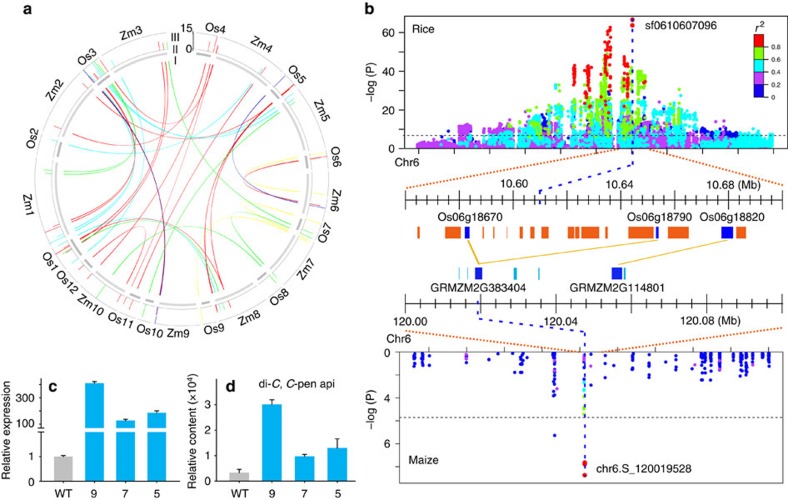
Comparative mGWAS between rice and maize. (**a**) The global view and relationships of comparative mGWAS results between rice and maize. I: oriented homologous loci between rice and maize. Links in colour represent homologous loci of different types of metabolites between rice and maize. Red: flavonoid; blue: nucleic acid; green: alkaloid, amino acid and fatty acid; yellow: polyamine and polyphenol; cyan: others and unknown. II: schematic diagram of chromosomes of rice and maize. The scale of chromosomes in maize is half of that in rice. III: bar plot of loci with candidate genes in rice and maize according to their −Log_10_(*P*) value. (**b**) Co-linear genomic regions and homologous loci (or genes) of di-*C*, *C*-pentosyl-apigenin between rice grains and maize kernels. *Os06g18670* and *Os06g18790* are homologous (or orthologous) to *GRMZM2G383404*. *Os06g18820* is homologous to *GRMZM2G114801*. Bar plots for the messenger RNA level of *Os06g18670* (**c**) and the content of di-*C*, *C*-pentosyl-apigenin (**d**) in transgenic individuals. WT: the transgenic background variety ZH11. The *P* value is calculated using the Student's *t* tests. Data are shown as the means±s.e.m., *n*=3.

Comparative linkage mapping among crop plants, such as wheat, maize and rice^27^, has revealed correspondence among QTLs in crop plants for traits such as seed size, shattering habit and flowering time and has been proposed to be a useful tool for predicting the loci of homologous major genes^28^,^45^. This concept was modified and extended to our mGWAS for candidate gene mining based on the co-regulation of targeted metabolic trait(s) between species (Supplementary Note 3). We performed comparative mGWAS between rice and maize by examining the genetic basis of the metabolic features detected in both species. A total of 420 (Supplementary Data 16) and 292 (Supplementary Data 17) loci were obtained for the 123 co-detected metabolic features in rice and maize (Supplementary Data 4), respectively. A search for homologous loci mapped by the same metabolites (or metabolites with similar structures) identified 42 loci underlying the 23 co-detected metabolic features between the two species (Fig. 3a and Supplementary Data 18). To test the significance of our GWAS overlaps, we used the randomization test described by Churchill *et al*.^46^ (Supplementary Note 3). The results indicated that on average, only 3.0 out of the 42 observed overlaps are likely due to chance alone (Supplementary Fig. 10). In addition to the three reported genes (Supplementary Note 3, Supplementary Fig. 11a,c and Supplementary Data 19), our comparative mGWAS revealed new candidates for the co-detected metabolites. For example, variation in the caffeic acid content mapped to the SNP sf0603183527 region on chr6 in rice and its homologous region at SNP SYNGENTA0813 in maize (Supplementary Fig. 11d and Supplementary Data 19), suggesting that these SNPs were indeed the loci responsible for the detected variations in both species. These two SNPs were located within *GRMZM2G127948* and 128 kb from *Os06g06980* in maize and rice (with a local linkage disequilibrium decay of 3 kb for maize and 150 kb for rice), respectively. Both loci encode putative *O*-methyltransferases, thus making them candidates for underlying caffeic acid in both species (Supplementary Fig. 11d and Supplementary Data 19). Additionally, we experimentally verified *Os11g25454* as the gene encoding apigenin glycosyltransferase, which underlies the variation of apigenin 7-*O*-glucoside in rice (Supplementary Fig. 11e,f and Supplementary Fig. 10), by using an *in vitro* enzyme assay. Similarly, the co-regulation of di-*C*, *C*-pentosyl-apigenin in both species facilitated the assignment of *Os06g18670* and *Os06g18790* (*E*=0 and 1E-155, respectively) as candidates underlying *C*-glycosyl-apigenin or its derivatives in rice (with a local linkage disequilibrium decay of 3 kb for maize and 50 kb for rice; Fig. 3b, Supplementary Fig. 12 and Supplementary Data 19). The expression of *Os06g18670* driven by the ubiquitin promoter resulted in the over-accumulation of di-*C*, *C*-pentosyl-apigenin in rice grains, whereas the overexpression of *Os06g18790* led to increased levels of a number of mono-*C*-hexosyl-apigenin derivatives. These findings support their annotation as apigenin *C*-glycosyltransferases (Fig. 3c,d, Supplementary Fig. 13). Twenty candidate genes were assigned using this approach (Supplementary Data 19).

### Dissecting complex traits by mGWAS and pGWAS

Traits related to the rice grain are particularly relevant for efforts to improve yield and end product^35^. To determine whether genetic analysis of the metabolome could facilitate the dissection of these complex traits, we measured six grain-related traits (hull colour, grain colour, grain width, grain thickness, grain length and 1000-grain weight) and performed a combined pGWAS (phenotypic genome-wide studies) and mGWAS analysis in rice grains (Supplementary Fig. 9). We assigned new candidate genes for these phenotypic traits (Supplementary Data 20) in addition to previously characterized genes and most of the candidate genes previously reported by other GWAS experiments (Supplementary Data 14). To further improve the dissection of these traits, links between phenotypic and metabolic traits were genetically inferred by evaluating common regions of genetic regulation or loci co-localization, taking advantage of the high resolution of the GWAS. To decrease possible false positives, we focused only on co-detected loci underlying metabolic traits that were highly correlated with the phenotypic traits (*r*>0.3 (ref. 24), *P*<1.5E-12, Pearson's correlation coefficient, Supplementary Data 21). By examining these highly resolved and co-localized loci in a functionally and biologically relevant manner, new loci with their candidate genes were assigned (Supplementary Data 14). In total, our parallel mGWAS and pGWAS identified 24 associated loci, including 17 new loci, for 6 grain-related traits (Table 1). For example, the correlation between *C*-hexosyl-chrysoeriol *O*-hexoside and hull colour (*r*=0.33, Pearson's correlation coefficient) and the co-localization between the two traits suggests that this metabolite might be involved in hull colouration. We assigned *Os02g41650* (encoding a putative phenylalanine ammonia lyase) as the candidate underlying this *C*-glycosyl flavone, owing to its high sequence identity (76% at the amino acid level) with *AtPAL1* and its co-expression with flavonoid biosynthetic genes such as *4CL*, *DFR* and *F3H* in rice. We also assigned *Os07g35060* (encoding F-box domain containing protein) as the candidate for grain thickness and *Os06g50400* (an expansin precursor) as the candidate underlying grain width. Detailed information about the associated loci and their candidate genes is provided in Supplementary Data 14.

**Figure 4:**
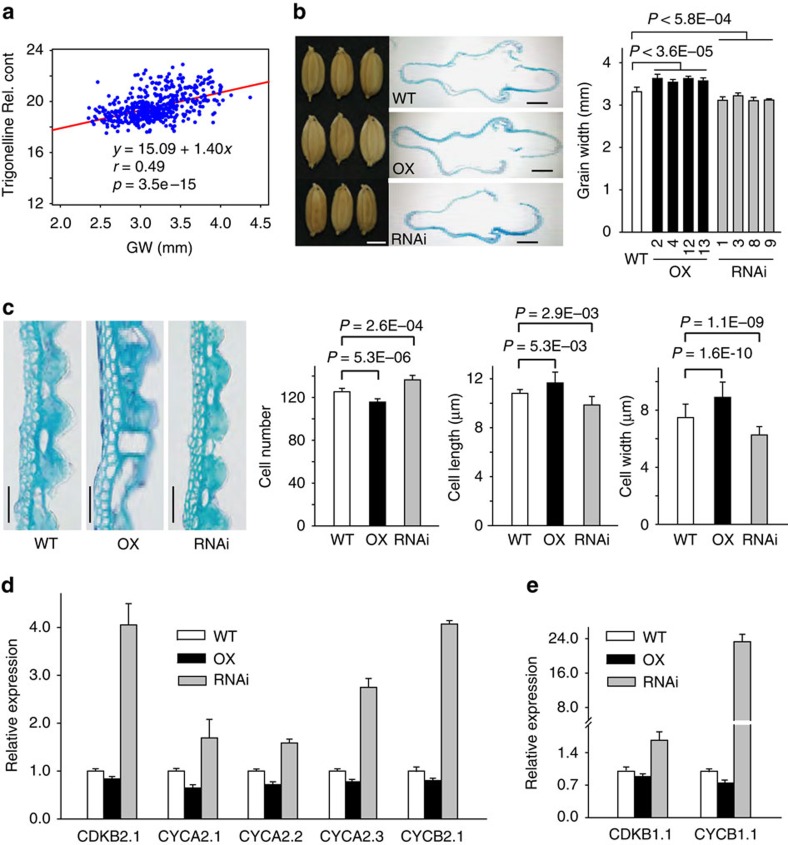
Evidence of metabotype-phenotype linkage. (**a**) Correlation between grain width (GW) and trigonelline content in 489 rice varieties. (**b**) Comparison of spikelet hull. Left: spikelet (scale bar, 3 mm). Middle: cross-section of spikelet hull (scale bar, 500 μm). Right: comparison of grain width. (**c**) Magnified view of spikelet hull cross-section from the box in **b**. Scale bar, 50 μm. Comparison of cell number, mean cell length and width in the outer parenchymal cell layers of spikelet hulls of WT, over-expression (OX) and RNA interference lines, respectively. (**d**) Transcript levels of genes associated with cell cycle regulation. (**e**) Transcript levels of genes involved in mitosis. The *r* value is based on the Pearson correlation coefficient. The *P* value is calculated using the Student's *t* tests. WT: the transgenic background variety ZH11. Data are shown as the means±s.e.m., *n*=3.

To experimentally validate the direct metabolite-phenotype association, we focused on the linkage between trigonelline levels and grain width due to the high correlation between these two traits (Fig. 4a). One of the major loci for the trigonelline levels mapped to a 35.3 Mb region (SNP sf0235265920) on chromosome 2 (*P*=2.8E-32, LMM, *n*=502). pGWAS showed that this locus was also responsible for the variation in grain width. Transgenic positive progeny (T2 generation) with overexpression of *Os02g57760* exhibited the over-accumulation of trigonelline and wider grains, whereas the T2 RNA interference plants showed the opposite phenotype (Fig. 4b and Supplementary Fig. 14). Therefore, we reasoned that *Os02g57760* and trigonelline were the quantitative trait gene and metabolite, respectively, underlying rice grain width.

In accordance with the data obtained from whole grains, similar results were observed for the length and width of the longitudinal epidermal cells of the outer glumes and inner glumes in the T1 *Os02g57760* transgenic lines, whereas the cell number exhibited the opposite trend (Fig. 4c). These data suggested that trigonelline regulates the grain width by promoting cell expansion. Similar phenotypes have been reported in tobacco BY-2 cells overexpressing SpCDC25 (ref. 47) and in tomato plants with down-regulated WEE1 (ref. 48), which results in a reduction in the mitotic cell length. Because trigonelline has been reported to induce G2 cell cycle arrest^42^, we analysed the expression of five genes putatively involved in the G2/M phase and two genes having important roles in mitosis, namely *CDKB2.1*, *CYCA2.1*, *CYCA2.2*, *CYCA2.3*, *CYCB2.1*, *CDKB1.1* and *CYCB1.1* (ref. 49; Fig. 4d,e). The transcript levels of these seven putative G2/M-phase genes were significantly downregulated and elevated in the over-expression (OX) and RNA interference plants, respectively, compared with wild type plants (Fig. 4e). These results suggest that trigonelline positively affects the grain width by elongating the G2 phase and the duration of the whole cell cycle. Further research is needed to obtain a detailed mechanistic understanding of the role of trigonelline in regulating grain width.

## Discussion

The importance of rice as the major diet supporting half of the world's population makes it an invaluable research target. Understanding the natural variation and genetic control of a wide spectrum of metabolites, including ones with nutritional and health-promoting importance such as amino acids and flavonoids, in rice grains has been furthered in this work and in previous studies^7^,^50^.

A large number of genetic studies have been performed to identify QTLs for a broad range of primary and/or secondary metabolites in both crops^6^,^11^,^12^,^51^,^52^,^53^ and non-crop plants^5^,^54^,^55^,^56^,^57^. Our current broader-scale profiling, based on these studies, but performed with a larger sample size and using more markers contributed to the identification of more mapped loci with an overall higher resolution. We demonstrated that the distinct genetically controlled natural variation of metabolism in different tissues is likely partially determined by allelic variation in genes that are expressed specifically or preferentially in individual tissues and encode enzymes responsible for metablolite biosynthesis (Fig. 1). Additionally, the joint analysis of individual metabolites across the two tissues via multivariate GWAS analysis using the MTMM approach^58^ makes it possible to distinguish association signals shared across the tissues from signals specific to one tissue (Supplementary Figs. 4 and 5 and Supplementary Data 12).

Despite the distinct genetic architecture for complex traits observed between rice and maize, representing selfing and outcrossing species^25^, respectively, our comparative mGWAS analysis indicated that the two plants likely share common genetic control strategies for certain metabolites (Supplementary Data 19). Taking advantage of the high resolution and the saturated SNPs in the maize and rice mGWASs^7^,^9^,^13^, respectively, we examined the ‘common' genetic loci that determine the levels of the same or similar metabolites not only to cross-validate the GWAS results in both species but also to facilitate the identification of new loci and the assignment of corresponding putative causative genes for these metabolic traits (Fig. 3 and Supplementary Data 19). However, this strategy is restricted to the ‘common' loci shared between plant species^28^,^45^.

Understanding the links between genotype and phenotype in Asian rice may aid efforts to improve world food supplies in terms of sustainability and reliability as well as quality and safety^25^,^35^. Investigations of the genetically inferred links between phenotypic and metabolic traits by QTL (or GWAS) co-localization based on linkage mapping have provided evidence for the genetic co-regulation of these traits^6^,^9^,^34^ and in some cases for the assignment of candidates underlying the interactions in model plants^33^. Benefitting from the relatively high resolution of GWAS compared with QTL analysis, the evaluation of common regions that affect both metabolic and phenotypic traits by parallel mGWAS and pGWAS has identified candidate biomarkers for traits such as grain colour and grain size (Supplementary Data 21). Moreover, we were able to generate testable hypotheses and experimentally further validate a role for *Os02g57760* (the nicotinic acid *N*-methyltransferase), in determining grain width (Fig. 4). This strategy may be applied to the dissection of the causative factors of phenotypic traits, particularly minor QTLs and metabolic composition.

In summary, the integrative approach described here is a powerful strategy for interactive rice functional genomics and metabolomics, which should help elucidate the overall genetic and biochemical regulation of metabolic and agronomic traits and lead to more rational and rapid genetic improvements in crops.

## Methods

### Plant material and growth conditions

A diverse worldwide collection of 502 *O. sativa* accessions including both landraces and elite varieties was obtained^16^. Information about the accessions, including variety name, country of origin, longitude and latitude origin and subpopulation identity is listed in Supplementary Data 1. The metabolite data set presented was based on 502 field-grown accessions from two years, 2012 and 2013. Plants were grown in a randomized complete-block design (including two rows of each accession and ten plants in each row) with two replicates for each year^59^. Two leaves were harvested from each of three randomly chosen plants at the five-leaf stage and pooled for leaf samples^16^. Mature seeds were randomly collected from 3 of the 17 remaining plants and pooled for metabolic profiling. In all, four sample sets (two years * two replications for each year) per accession were used for metabolomics studies.

Rice plants examined under field conditions were grown during the normal rice-growing seasons in the Experimental Station of Huazhong Agricultural University (Wuhan, China). All seeds were planted in a seedbed in mid-May and transplanted to the field in mid-June. The planting density was 16.5 cm between plants in a row, with the rows 26 cm apart. Field management, including irrigation, fertilizer application and pest control are essentially followed normal agricultural practice.

### Metabolite profiling

A liquid chromatography-electrospray ionization-tandem mass spectrometry system was used for the relative quantification of widely targeted metabolites in dried rice grain samples^60^. The dried rice grain was crushed using a mixer mill (MM 400, Retsch) with a zirconia bead for 1.5 min at 30 Hz, 100 mg dried powder was weighted and extracted overnight at 4 °C with 1.0 ml pure methanol (or 70% aqueous methanol) containing 0.1 mg l^−1^ lidocaine (internal standard) for lipid-solubility metabolites (or water-solubility metabolites). Quantification of metabolites was carried out using a scheduled multiple reaction monitoring method^60^. The relative signal intensities of metabolites were normalized by first dividing them by the intensities of the internal standard (lidocaine, 0.1 mg l^−1^; ref. 7) and then log 2 transforming them for further normalization to improve the normality. A data matrix containing the 837 relative intensities of metabolites from 2008 runs (502 accessions * four sample sets) was produced for the rice population (Supplementary Data 3). The m-trait data of the association panel are the mean values of the four biological sample sets for the liquid chromatography–mass spectrometry as shown below: *P*_*m*,l_=1/4(*P*_*m*,l,1_+*P*_*m*,l,2_+*P*_*m*,l,4_+*P*_*m*,l,4_), where *P*_*m*,l_ represents the m-trait data for metabolite *m* (*m*=1, 2, 3, ..., 837 in grain) in accession l (l=1, 2, 3, ..., 502), and *P*_*m*,l,1_, *P*_*m*,l,2_, *P*_*m*,l,3_ and *P*_*m*,l,4_ are the normalized metabolite levels determined in the four sample sets, respectively.

### Genome-wide association analyses

Sequence data were obtained from the website RiceVarMap (http://ricevarmap.ncpgr.cn)^40^. Only SNPs with an MAF≥0.05 and the number of varieties with a minor allele≥6 in a (sub) panel were used to perform the mGWAS. There are 2,767,191, 1,857,866 and 3,916,415 SNPs used in GWAS for subpopulations of *Indica*, *Japonica* and the whole panel, respectively. Population structure was modelled as a random effect in LMM using the kinship (K) matrix. We performed GWAS using LMM provided by FaST-LMM program^61^. Two different genome-wide thresholds (significant and suggestive)^62^ were set to define associations, using a ‘modified' Bonferroni correction described by Li *et al*.^63^ in which the total SNPs for threshold calculation was replaced by the effective number of independent SNPs (Me). The calculated genome-wide significant threshold, based on the original Bonferroni calculation of 0.05/Me, were 6.6E-8, 8.7E-8, and 2.0E-7 (LMM, *n*=502) for *All*, *Indica* and *Japonica*, respectively^62^. The calculated genome-wide suggestive threshold, based on the original Bonferroni calculation of 1/Me, were 1.3E-6, 1.8E-6 and 4.1E-6 (LMM, *n*=502) for *All*, *Indica* and *Japonica*, respectively^62^.

### Statistical analysis

The coefficient of variation^19^ values were independently calculated for each metabolite (using the mean of the four sample sets of the normalized metabolic data) as below: σ/μ, σ and μ represent the s.d. and the mean of each metabolite relative intensity in the population, respectively. The broad-sense heritability (H^2^) was estimated using mixed effects model^12^ with random effects for genotype (502 accessions), environment (years 2012 and 2013), and genotype-environment interactions. We used the lmer function from the lme4 package^64^ in the R environment.

Linkage disequilibrium was estimated using standardized disequilibrium coefficients (D') and squared allele-frequency correlations (*r*^*2*^) for pairs of SNP loci according to the TASSEL software program (http://www.maizegenetics.net/tassel). Linkage disequilibrium plots were generated in Haploview, indicating the *r*^*2*^ values between pairs of SNPs (white, *r*^*2*^=0, shades of grey, 0<*r^2^<*1 and black, *r*^*2*^=1, Pearson's correlation coefficient).

### Gaussian graphical modelling

GGM, an undirected probabilistic graphical model estimating the conditional dependence between variables, is based on pairwise Pearson correlation coefficients conditioned against the correlation with all other metabolites^65^. A full data matrix was constructed from 502 samples for the different subgroups (all, *indica* and *japonica*) and 587 metabolites for the GGM calculation. GeneNet package 1.2.8 (from the CRAN, http://www.cran.r-project.org/) was used to estimate the *P*-correlation and assess the significance of the edges between metabolites. A significant *P* value<2.9E-07 (0.05/171,991) with an absolute partial correlation cutoff of *P*=0.05 was applied to filter the results. In total, 1,464 metabolite pairs were used to construct a metabolic network with the software Cytoscape (3.0.2).

### Homologous loci detection and gene model identification

The co-detected metabolic traits in both species were used to filter out loci through mGWAS in rice and maize grain, respectively. The calculated genome-wide threshold was set at *P*=1.8E-06 (MLM, *n*=339) for maize^15^ and *P*=1.3E-06, 1.8E-06 and 4.1E-06 (LMM, *n*=502) for whole panel, *indica* and *japonica* of rice, respectively^16^. The sequence alignment analysis was based on a VISTA sequence alignment algorithm program^66^ between the rice genome (Nipponbare, MSU version 6.1) and the maize kernel genome (B73, RefGen_v2). A BLAST search was performed on any maize peptide sequence against the internationally available rice databases. Expression profile data were obtained from CREP (http://crep.ncpgr.cn/crep-cgi/home.pl). The visualization of homologous blocks and significant loci with functional genes was performed with Circos^67^. To test the significance of our GWAS overlaps, we adopted the randomization test of Churchill *et al*.^46^ to determine the proportion of overlaps expected to occur by chance (Supplementary Note 3).

### Phenotyping

Fully filled grains were used to measure the grain length, width, thickness and weight. Twenty randomly chosen grains were lined up length-wise to measure the grain length using an electronic digital caliper, then arranged by breadth to measure the grain width. The grain thickness was determined individually for each grain using an electronic digital caliper. Finally, the values were averaged and used as the measurements. The grain weight was initially obtained by weighing a total of 100 grains, then converting the average of three independent repeats to the 1,000-grain weight, which is a commonly used scale for yield evaluation. The hull colour and seed colour were scored on a scale of 1–4 (white, yellow, red and black).

### Phylogenetic analysis different gene families

The amino acid sequences were aligned using the CLUSTALW (version 1.83) program. The neighbour-joining tree was constructed using aligned full-length amino acid sequences (MEGA5). Bootstrap values from 1,000 replicates are indicated at each node. Bar=0.1 amino acid substitutions per site.

### Rice transformation and expression analyses

The over-expression constructs of *Os02g57600*, *Os06g18670* and *Os06g18790* were generated by directionally inserting the full complementary DNA (cDNAs) from Zhenshan 97 first into the entry vector DONR207 and then into the destination vector pJC034 uses the Gateway recombination reaction (Invitrogen). Primers used in this study are shown in Supplementary Data 23. For each constructs, at least three independent over-expression plants were selected for the targeted metabolites analysis.

We isolated total RNA from rice using an RNA extraction kit (TRIzol reagent; Invitrogen) according to the manufacturer's instructions. The first-strand cDNA was synthesized using 3 μg RNA and 200U M-MLV (Invitrogen) reverse transcriptase according to the manufacturer's protocol. The expression measurements were obtained using the relative quantification method.

### Expression of candidate genes

Full-length cDNA of candidate genes (*Os11g42370*, *Os11g25454* and *Os04g11970*) were amplified with the primers using cDNA from Nipponbare as a template. The expression constructs of *Os11g42370* (*Os11g25454* and *Os04g11970*) were generated by directionally inserting the full cDNA into the entry vector *pDONR207* (Invitrogen) and then Error-free clones was into the expression vector *pDEST17* (or *pDEST15*) by attL × attR (LR) recombination (Invitrogen). Recombinant proteins were expressed in BL21 (DE3) cells (Novagene) following induction by addition of 0.4 mM isopropylthiogalactoside and growing continually for 12 h at 16 °C. Cells were harvested and pellets were frozen at −80 °C. Pellets were re-suspended in 50 mM sodium phosphate buffer (pH 7.8) and lysed by sonication. The crude extract was collected and clarified by centrifugation at 12,000*g* for 15 min at 4 °C and supernatant of the crude enzyme was stored at −80 °C.

### Candidate genes assay

The enzyme reactions *in vitro* assay for the biosynthesis of *N*-cinnamoyltrypamine, *N*-benzoyltrypamine and *N*-benzoylserotionin were performed in a total volume of 100 μl containing 200 μM Cinnmoyl-*CoA* (Benzyol-*CoA*), 1 mM serotonin (trypamine) and 5 μl supernatant protein in potassium phosphate buffer (100 mM, pH 7.4) was incubated for 1 h at 37 °C. For *Os11g25454*, a total volume of 100 μl containing 100 μM apigenin substrate, 1 mM uridine diphosphate glucose (UDP glucose), 5 mM Mg^2+^ and 5 μl supernatant protein in potassium phosphate buffer (100 mM, pH 6.8) was incubated for 1 h at 37 °C. For *Os04g11970*, the standard *in vitro* assay for the biosynthesis of *O*-methylapigenin *C*-hexoside was performed in a total volume of 100 μl containing 100 μM AdoMet SAM (sigma) and 50 μM apigenin 6-*C*-glucoside in 10 mM sodium phosphate buffer (pH 7.8). After incubating at 37 °C for 1 h, the reaction was stopped by adding 200 μl of ice-cold 0.5% trifluoroacetic acid. The reaction mixture was then filtered through a 0.2 μm filter (Millipore) before being used for liquid chromatography–mass spectrometry analysis.

### Sectioning and microscopy

Paraffin sections were made according to the method of Yao *et al*.^68^. Samples were whole stained with Ehrlich's hematoxylin for 2 days before dehydration and restrained with toluidine blue after section acquisition. Cellular observation was realized by using Olympus BX61 (Olympus, Japan).

### Source data sets

Information regarding the source of published data sets^15^,^16^ used in Supplementary Tables 3, 8 and 9 and Supplementary Data 2 and 3 are shown in Supplementary Data 22.

### Data availability

The authors declare that all other data supporting the findings of this study are available within the manuscript and its supplementary information files or are available from the corresponding author upon request.

## Additional information

**How to cite this article:** Chen, W. *et al*. Comparative and parallel genome-wide association studies for metabolic and agronomic traits in cereals. *Nat. Commun.* 7:12767 doi: 10.1038/ncomms12767 (2016).

## Supplementary Material

Supplementary Information Supplementary Figures 1 – 14, Supplementary Notes 1 – 3 and Supplementary References

Supplementary Data 1 502 rice varieties list.

Supplementary Data 2 Scheduled MRM transitions for metabolite analysis.

Supplementary Data 3 Data matrix of related metabolites and phenotypes.

Supplementary Data 4 Metabolite list with significant H^2^.

Supplementary Data 5 CV and H^2^ of some detected metabolites.

Supplementary Data 6 Correlation between the detected metabolites.

Supplementary Data 7 Metabolite relative content between different subgroups.

Supplementary Data 8 1489 significant SNPs detected in at least one population.

Supplementary Data 9 476 loci detected in at least one population.

Supplementary Data 10 Summary of significant associations identified.

Supplementary Data 11 6366 suggestive SNPs detected in at least population.

Supplementary Data 12 Compare metabolite relative accumulation between tissues.

Supplementary Data 13 Compare summary results of mGWAS between tissues.

Supplementary Data 14 The candidate gene list of mGWAS and pGWAS.

Supplementary Data 15 The GGM results of rice grain in different populations.

Supplementary Data 16 420 loci in rice for co-detected metabolites in both species.

Supplementary Data 17 292 loci in maize for co-detected metabolites in both species.

Supplementary Data 18 42 loci for 23 metabolites (or metabolites with similar structure) were detected simultaneously in both species.

Supplementary Data 19 Candidate gene list from the comparative mGWAS.

Supplementary Data 20 New loci and candidate genes for phenotypic traits.

Supplementary Data 21 Correlation between phenotypic traits and metabolites.

Supplementary Data 22 Source datasets information.

Supplementary Data 23 Primers used in this study.

## Figures and Tables

**Table 1:** Summary of 32 candidate genes were newly disclosed by examining the mGWAS data from rice grain.

Metabolite	Associated SNP	M-*P* value*	P-trait	P-*P* value†	Cor‡	Candidate gene	Description
Cystathionine	sf0218685490	1.20E-07				*Os02g31200*	Esterase
Asparagine	sf0310123005	5.70E-07				*Os03g18130*	Asparagine synthetase
LPC (1-acyl16:1)	vf0131875915	4.44E-07				*Os01g55360*	Cytidylyltransferase
Sinapoylcholine	sf0629727750	1.89E-09				*Os06g49050*	hAT
Trigonelline	sf0235364705§	2.78E-32	GW	1.2E-08	0.40	*Os02g57760*	*O*-methyltransferase
Trigonelline	sf0314995948	5.63E-08	GW	7.5E-08	0.40	*Os03g26200*	*O*-methyltransferase
Trigonelline	sf0630519510	1.63E-16	GW	1.3E-08	0.40	*Os06g50400*	Expansin precursor
Trigonelline	sf0707312584	8.93E-11	GW	6.6E-06	0.40	*Os07g12780*	Cyclin
Trigonelline	sf0523433859	2.59E-16	GT	9.8E-11	0.38	*Os05g39990*	Expansin precursor
Trigonelline	sf0721002801	5.94E-08	GT	6.3E-06	0.38	*Os07g35060*	OsFBX238
Trigonelline	sf1108644317	9.84E-18				*Os11g15300*	*O*-methyltransferase
*C*-hex-chr *O*-hex	sf0224981507§	2.39E-08	HC	2.2E-06	0.33	*Os02g41650*	PAL
Cya *O*-rut	sf0424349335	8.17E-12	HC	6.2E-07	0.50	*Os04g41350*	Amino acid transporter
Del *O*-hex der	sf0424523684	1.69E-10	HC	4.0E-09	0.61	*Os04g41680*	Endochitinase A
Peo *O*-hex	sf1005351606	1.65E-10	HC	1.7E-07	0.40	*Os10g09860*	Chalcone synthase
Catechin	sf0405211891	6.25E-10	SC	8.8E-09	0.91	*Os04g09720*	OsSCP22
Peo *O*-rut	sf0423865941	9.05E-10	SC	9.4E-07	0.60	*Os04g40470*	Cytochrome P450
Peo *O*-hex	sf0428130219	1.97E-12	SC	3.5E-08	0.31	*Os04g47720*	UGT
Di-catechin	sf0519583338	1.43E-18	SC	6.8E-15	0.86	*Os05g33430*	Xyloglucanase inhibitor
*C*-pen-api *O*-rut	sf0520731687	1.73E-07	SC	2.3E-09	−0.37	*Os05g35010*	Cytochrome P450
Catechin	sf0706686370	2.26E-13	SC	1.9E-13	0.91	Os07g11440	Chalcone synthase
Catechin	sf1223103558	1.15E-10	SC	2.1E-07	0.91	*Os12g37690*	MYB
Eri *O*-mhex	sf0129468555	3.39E-08				*Os01g51260*	MYB
Pel *O*-hex	sf0132150258	2.10E-12				*Os01g55830*	GST
Peo *O*-hex	vf0235263818	1.21E-12				*Os02g57580*	Anthocyanin permease
Del *O*-rut	sf0304428849	2.63E-07				*Os03g08600*	UGT
*O*-methylapi-*C*-hex	vf0406561691||	6.74E-47				*Os04g11970*	*O*-methyltransferase
*C*-pen-api *O*-rut	sf0524320092	3.25E-52				*Os05g41645*	Chalcone synthase
Tri *O*-hex-hex	sf0526155386	9.84E-19				*Os05g45150*	UGT
*C*-hex-lut *O*-couhex	sf1008424002	6.59E-08				*Os10g16974*	Cytochrome P450
Chr *O*-hex-rut	sf1111581156	3.90E-13				*Os11g20080*	*O*-methyltransferase
Tri *O*-gluc-*O*-hex	sf1222982501	3.87E-18				*Os12g37510*	UGT

Metabolites abbreviations: Cya *O*-rut, cyanidin *O*-rutinoside; *C*-hex-chr *O*-hex, *C*-hexosyl-chrysoeriol *O*-hexoside; Chr *O*-ferhex-*O*-hex, chrysoeriol *O*-feruloylhexosyl-*O*-hexoside; Chr *O*-hex-rut, chrysoeriol *O*-hexosyl-*O*-rutinoside; Del *O*-rut, delphinidin *O*-rutinoside; Del *O*-hex der, delphinidin *O*-hexoside derivative; Eri *O*-mhex, eriodictyol *O*-malonylhexoside; *C*-hex-api *O*-couhex, *C*-hexosyl-apigenin *O*-*p*-coumaroylhexoside; *O-*methylapi-*C*-hex, *O*-methylapigenin-*C*-hexoside; Peo *O*-rut, peonidin *O*-rutinoside; *C*-pen-api *O*-rut, *C*-pentosyl-apigenin *O*-rutinoside; Peo *O*-hex, peonidin *O*-hexoside; *C*-pen-api *O*-rut, *C*-pentosyl-apigenin *O*-rutinoside; Pel *O*-hex, pelargonidin *O*-hexoside; Tri *O*-hex-hex, tricin *O*-hexosyl-*O*-hexoside; Tri *O*-gluc-*O*-hex, tricin *O*-glucuronide-*O*-hexoside. Phenotype abbreviations: GW, grain width; GT, grain thickness; GST, glutathione S-transferase; HC, hull colour; LPC, lysophosphatidylcholine; hAT, hAT transposon superfamily protein; PAL, phenylalanine ammonia lyase; SC, seed colour.

^*^P value of the corresponding metabolic trait calculated by LMM.

^†^*P* value of the corresponding phenotypic trait calculated by LMM.

^‡^The correlation between metabotype and phenotype.

^§^SNP introducing initiation codon.

^||^2-bp deletion.
