# Supplementary material for: Comparative and parallel genome-wide association studies for metabolic and agronomic traits in cereals
Source: Nat Commun. 2016 Oct 4;7:12767. doi: 10.1038/ncomms12767 (PMC5059443; doi:10.1038/ncomms12767)
Supplement: Supplementary Information — Supplementary Figures 1 – 14, Supplementary Notes 1 – 3 and Supplementary References [file ncomms12767-s1.pdf]

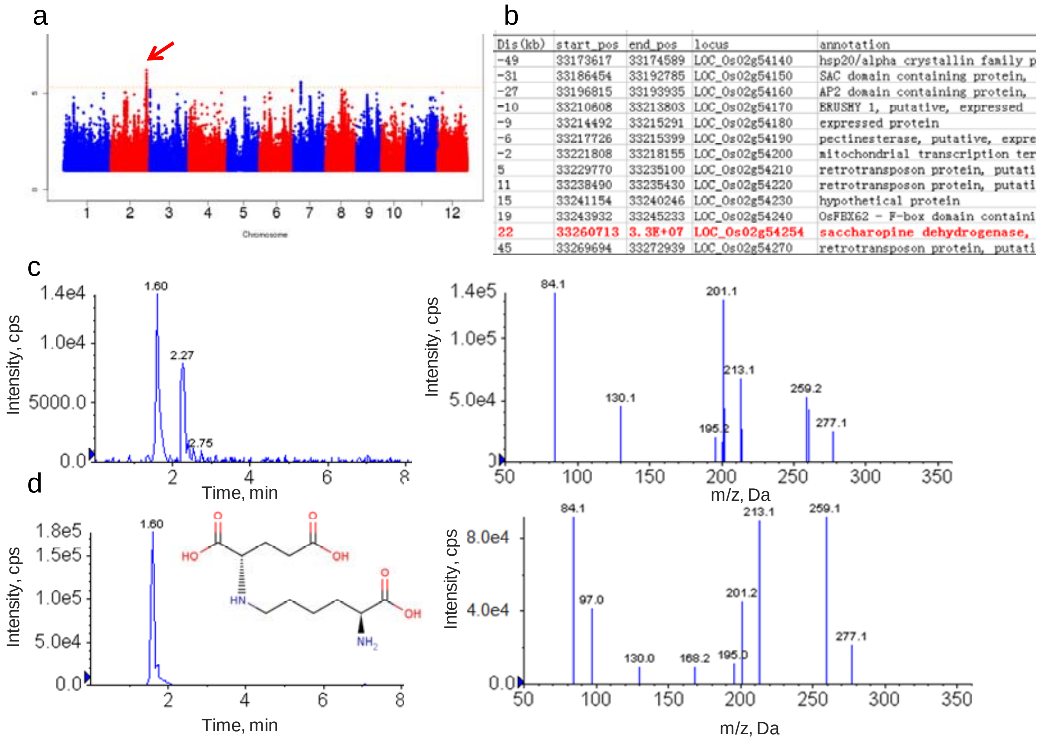

Dis(kb)	start_pos	end_pos	locus	annotation
-49	33173617	33174589	LOC_Os02g54140	hsp20/alpha crystallin family p
-31	33186454	33192785	LOC_Os02g54150	SAC domain containing protein,
-27	33196815	33193935	LOC_Os02g54160	AP2 domain containing protein,
-10	33210608	33213803	LOC_Os02g54170	BRUSHY 1, putative, expressed
-9	33214492	33215291	LOC_Os02g54180	expressed protein
-6	33217726	33215399	LOC_Os02g54190	pectinesterase, putative, expre
-2	33221808	33218155	LOC_Os02g54200	mitochondrial transcription ter
5	33229770	33235100	LOC_Os02g54210	retrotransposon protein, putati
11	33238490	33235430	LOC_Os02g54220	retrotransposon protein, putati
15	33241154	33240246	LOC_Os02g54230	hypothetical protein
19	33243932	33245233	LOC_Os02g54240	OsFBX62 - F-box domain containi
22	33260713	3. 3E+07	LOC_Os02g54254	saccharopine dehydrogenase,
45	33269694	33272939	LOC_Os02g54270	retrotransposon protein, putati

Supplementary Figure 1 Saccharopine identification facilitated by GWAS results.

(**a**) Manhattan plot displaying the GWAS result of the content of mr208 and the strongest association pointed by arrow in red. (**b**) The strongest association between SNP sf0233224406 is 22 kb away from *OsLKR*, the gene encoding saccharopine dehydrogenase, suggesting mr208 could be saccharopine. (**c**) Mr208 detected with MRM transition 277/84 in rice grain sample and (**d**) mr208 was confirmed as saccharopine by comparing the RT and fragmentation pattern with the commercial standard.

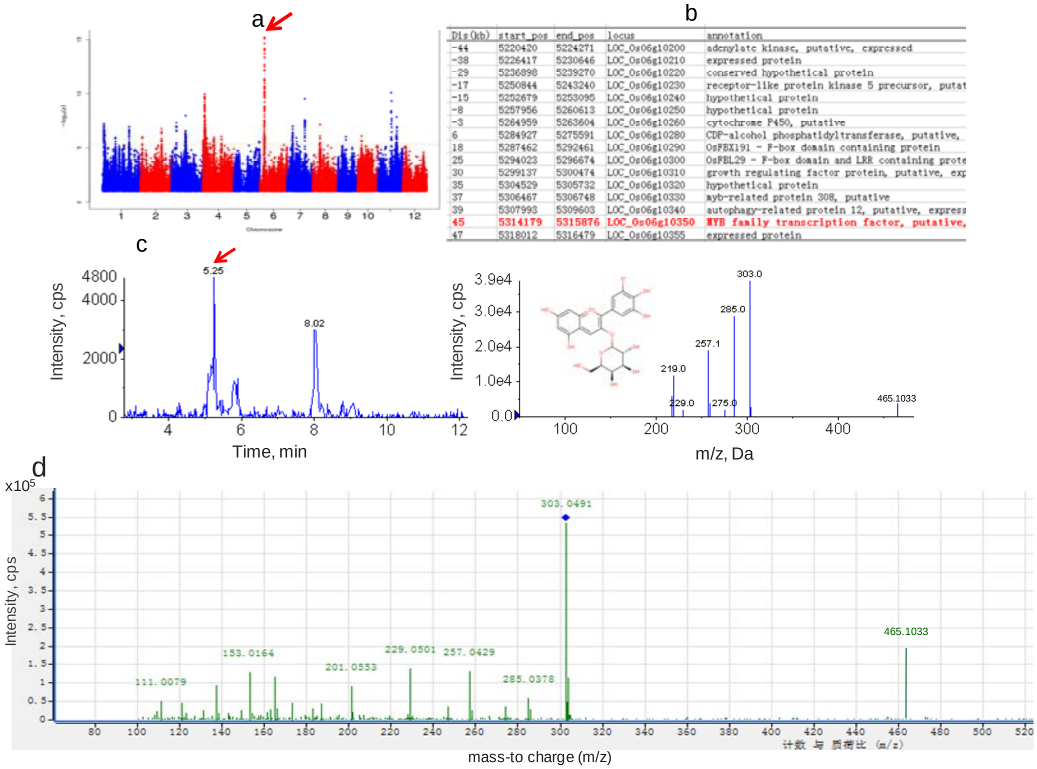

Supplementary Figure 2 Delphinidin 3-*O*-glucoside identification facilitated by GWAS results.

(**a**) Manhattan plot displaying the GWAS result of the content of mr063 and the strongest association pointed by arrow in red. (**b**) The strongest association between SNP sf0605268699 is 45 kb away from *OsC1*. (**c**) Mass peak (RT=5.2min) and spectrum of delphinidin 3-*O*-glucoside in rice grain sample (**d**) delphinidin 3-*O*-glucoside was confirmed by high resolution mass MS and MS/MS spectral pattern.

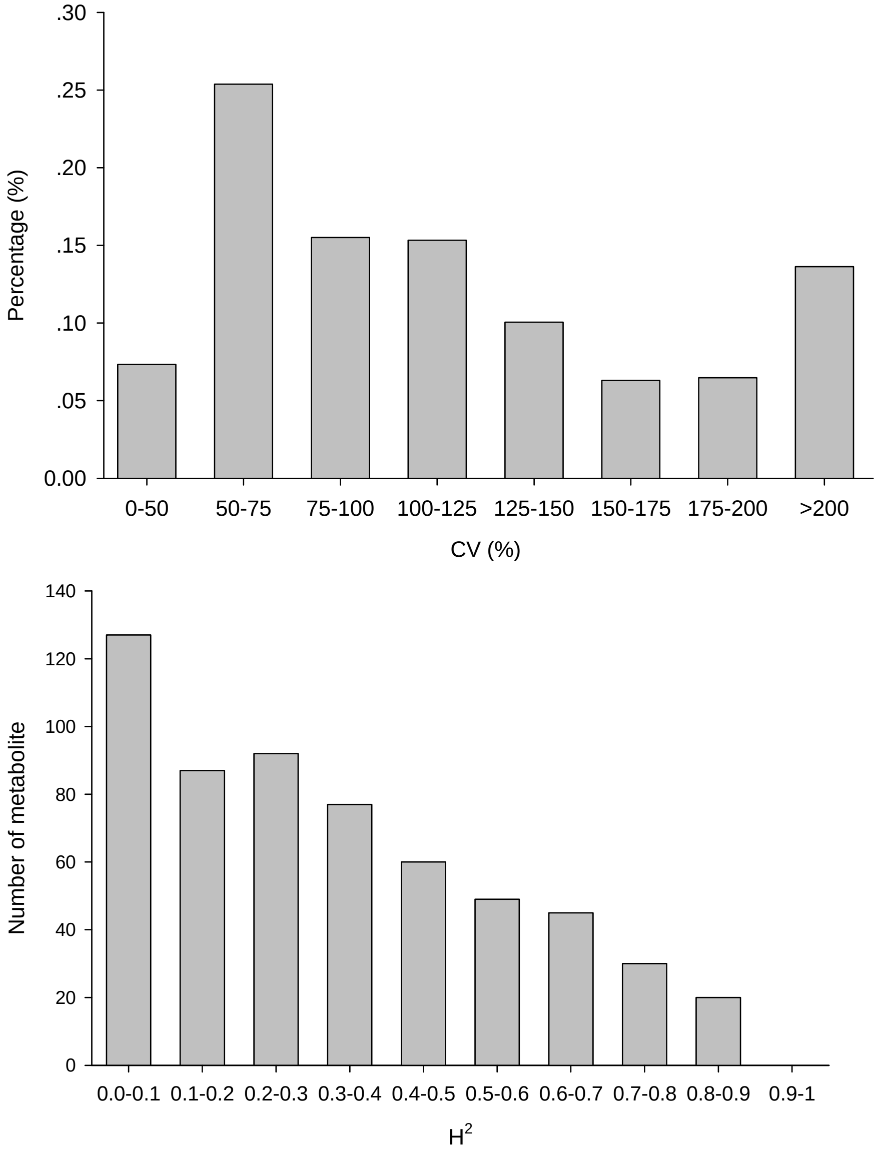

Supplementary Figure 3 The coefficients of variation (CV) and the broad-sense heritability (H^2) results for each metabolite.

(**a**) Distribution of the phenotypic coefficients of variation (CV) of metabolic traits (n = 587). (**b**) Distribution of broad-sense heritability (H^2) of metabolic traits (P < 0.05, two-way ANOVA) detected in the association panel.

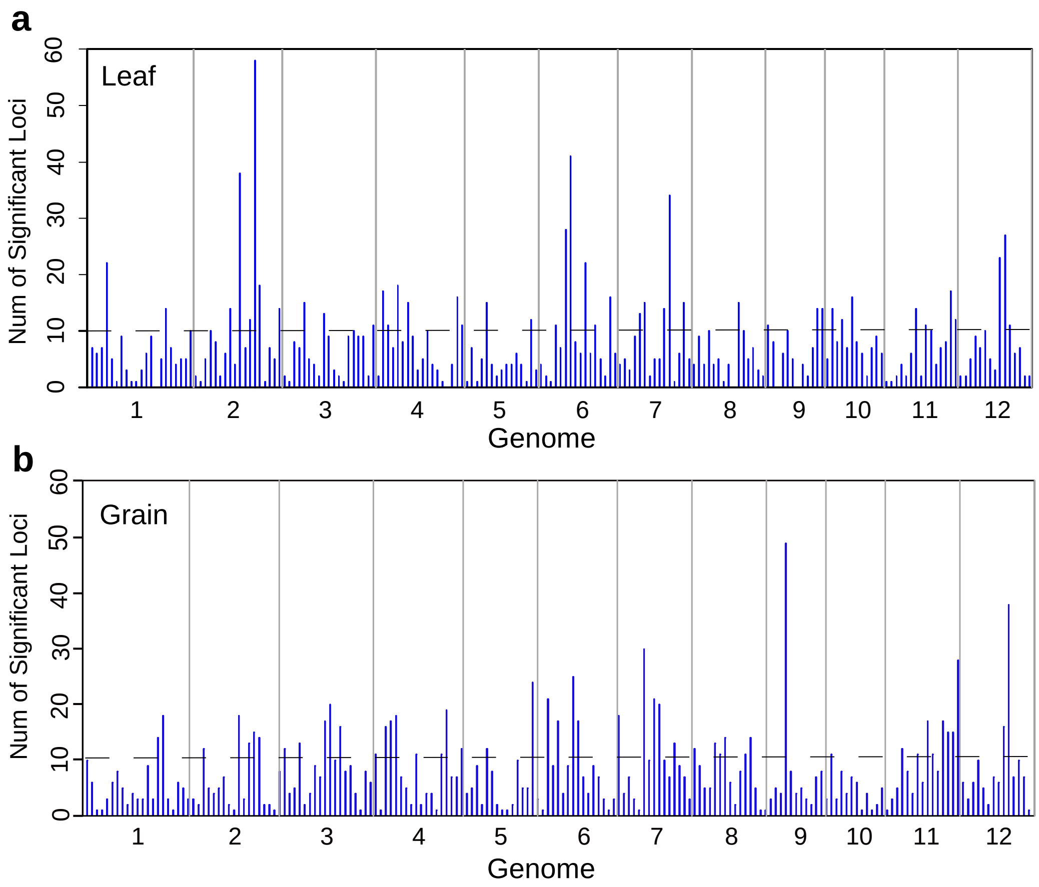

Supplementary Figure 4 Statistics of the number of the significant association for the mGWAS results in rice leaf (**a**) and rice grain (**b**).

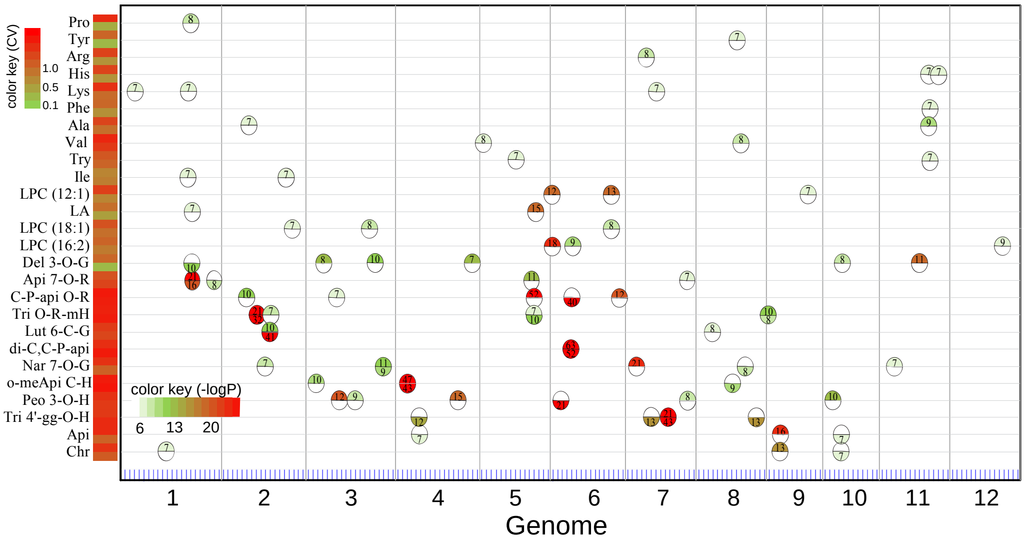

Supplementary Figure 5 Comparing the association for some same metabolites between rice grains (up) and rice leaf (down).

Pro, proline; Tyr, tyrosine; Arg, arginine; His, histidine; Lys, lysine; Phe, phenylalanine; Ala, alanine; Val, valine; Try, tryptamine; Ile, isoleucine; LPC (12:1), lysophosphatidyl choline (12:1); LA, linoleic acid; LPC(18:1), lysophosphatidyl choline(18:1); LPC(16:2), lysophosphatidyl choline(16:2); Api 7-*O*-G, apigenin 7-*O*-glucoside; Api 7-*O*-R, apigenin 7-*O*-rutinoside; *C*-P-api *O*-R, *C*-pentoside-apigenin *O*-rutinoside; Tri *O*-R-mh, tricin *O*-rutinoside-malonylhexoside; Lut 6-*C*-G, luteolin 6-*C*-glucoside; di-*C*,*C*-P-api, di-*C*,*C*-pentosyl-apigenin; Nar 7-*O*-G, naringenin 7-*O*-glucoside; o-meApi *C*-P, *O*-methylapigenin *C*-pentoside; Peo 3-*O*-H, peonidin 3-*O*-hexoside; Tri 4’-gg-*O*-H, Tricin 4'-*O*-(β-guaiacylglyceryl) ether *O*-hexoside; Api, apigenin; Chr, chrysoeriol.

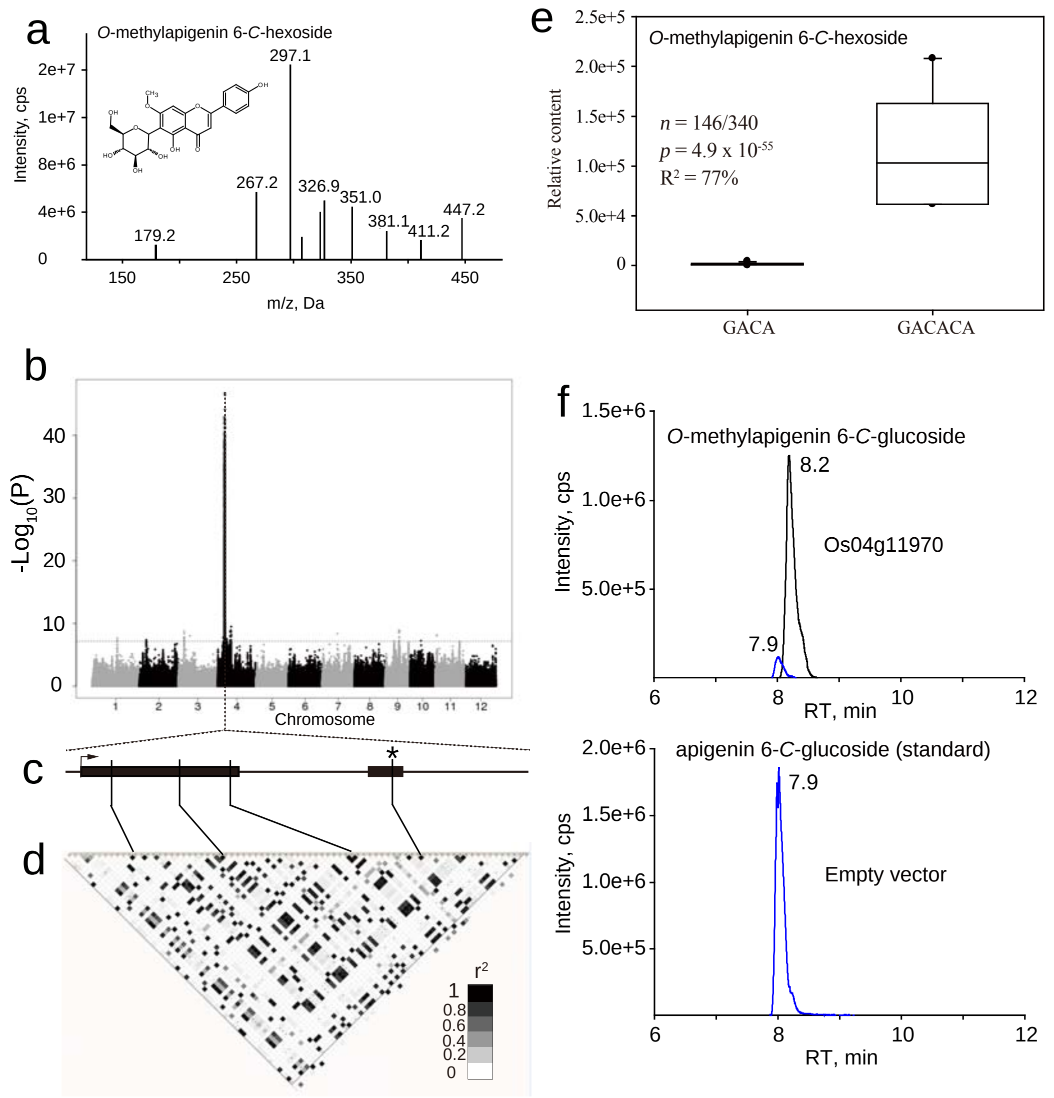

Supplementary Figure 6 Functional annotation of *Os04g11970* and the assignment of associated sites.

(**a**) Structure and LC-MS/MS fragmentation of *O*-methylapigenin 6-*C*-hexoside. Structure and the major fragments of *O*-methylapigenin 6-*C*-hexoside are shown. (**b**) Manhattan plot displaying the GWAS result of the content of *O*-methylapigenin 6-*C*-hexoside. (**c**) Gene model of *Os04g11970*. Filled black box represents coding sequence. The grey

vertical lines mark the polymorphic sites identified by high-throughput sequencing, and the stars represent the associated sites. (**d**) A representation of the pair-wise r^2 value (a measure of LD) among all polymorphic sites in *Os04g11970*, where the darkness of the color of each box corresponds to the r^2 value according to the legend. (**e**) Box plot indicate *O*-methylapigenin 6-*C*-hexoside content; plotted as a associated site at Chr4. vf0406561691. (**f**) LC-MS chromatograms of *in vitro* enzyme assays showing the enzyme activity of recombinant Os04g11970 (up). Protein extract from *E. coli* containing pDEST15 empty vector were used as a negative control (down).

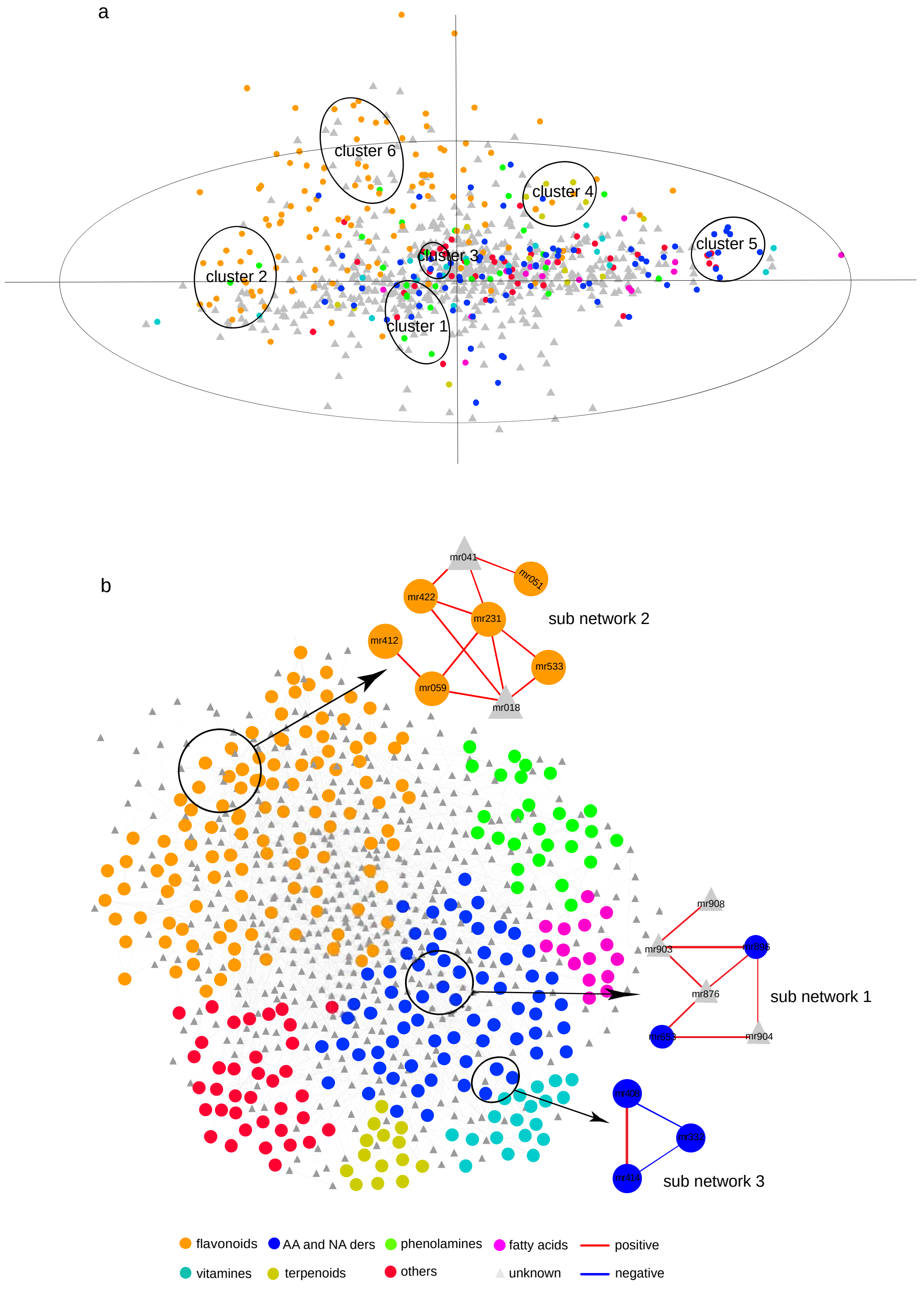

Supplementary Figure 7 The PCA (**a**) and GGM (**b**) results of rice grain.

AA and NA ders, amino acid and nucleic acid derivatives.

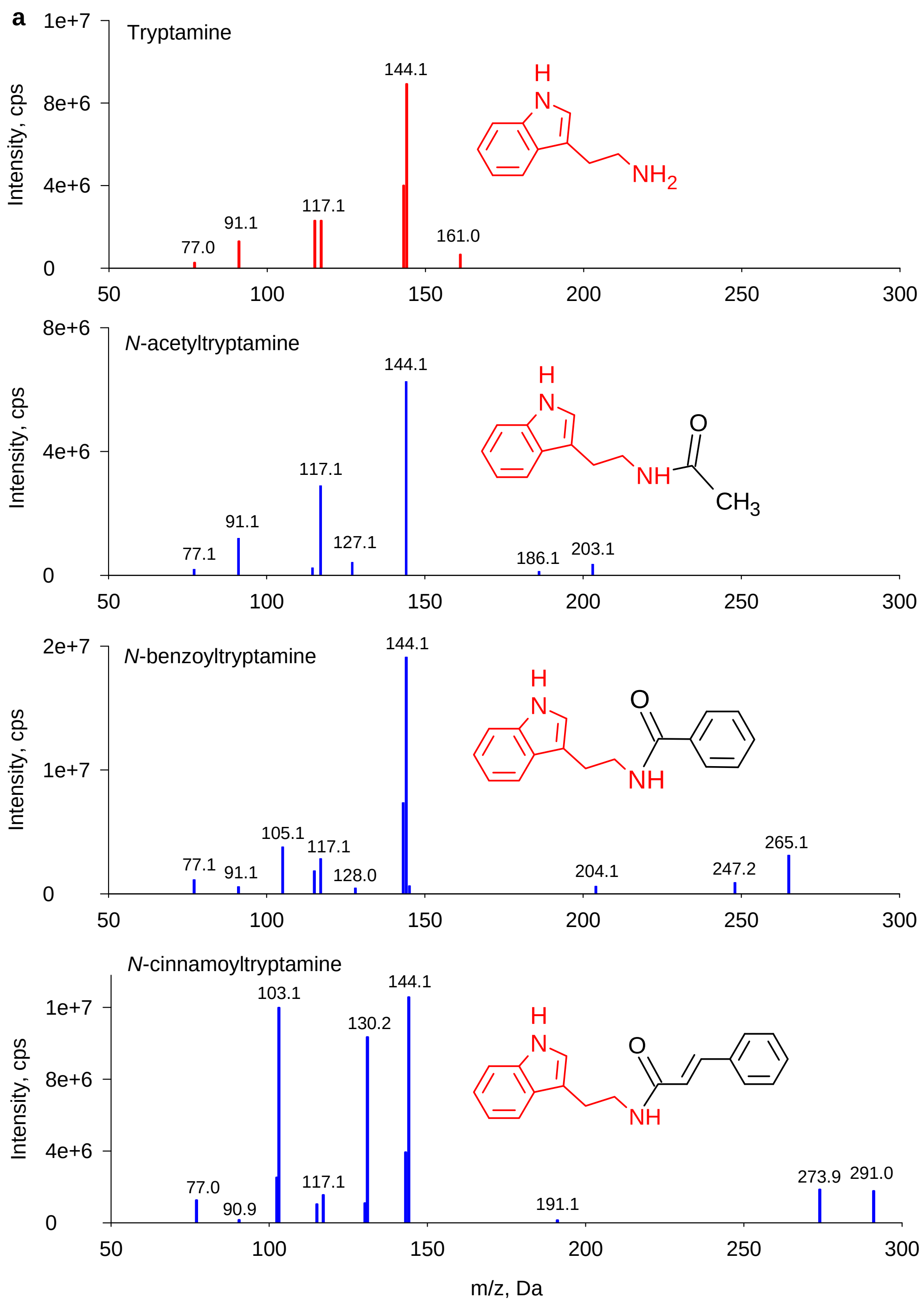
a
Tryptamine
N-acetyltryptamine
N-benzoyltryptamine
N-cinnamoyltryptamine
Intensity, cps
m/z, Da
144.1
117.1
91.1
77.0
161.0
127.1
186.1
203.1
105.1
128.0
204.1
247.2
265.1
103.1
130.2
90.9
191.1
273.9
291.0
NH2
NH
CH3
O

83

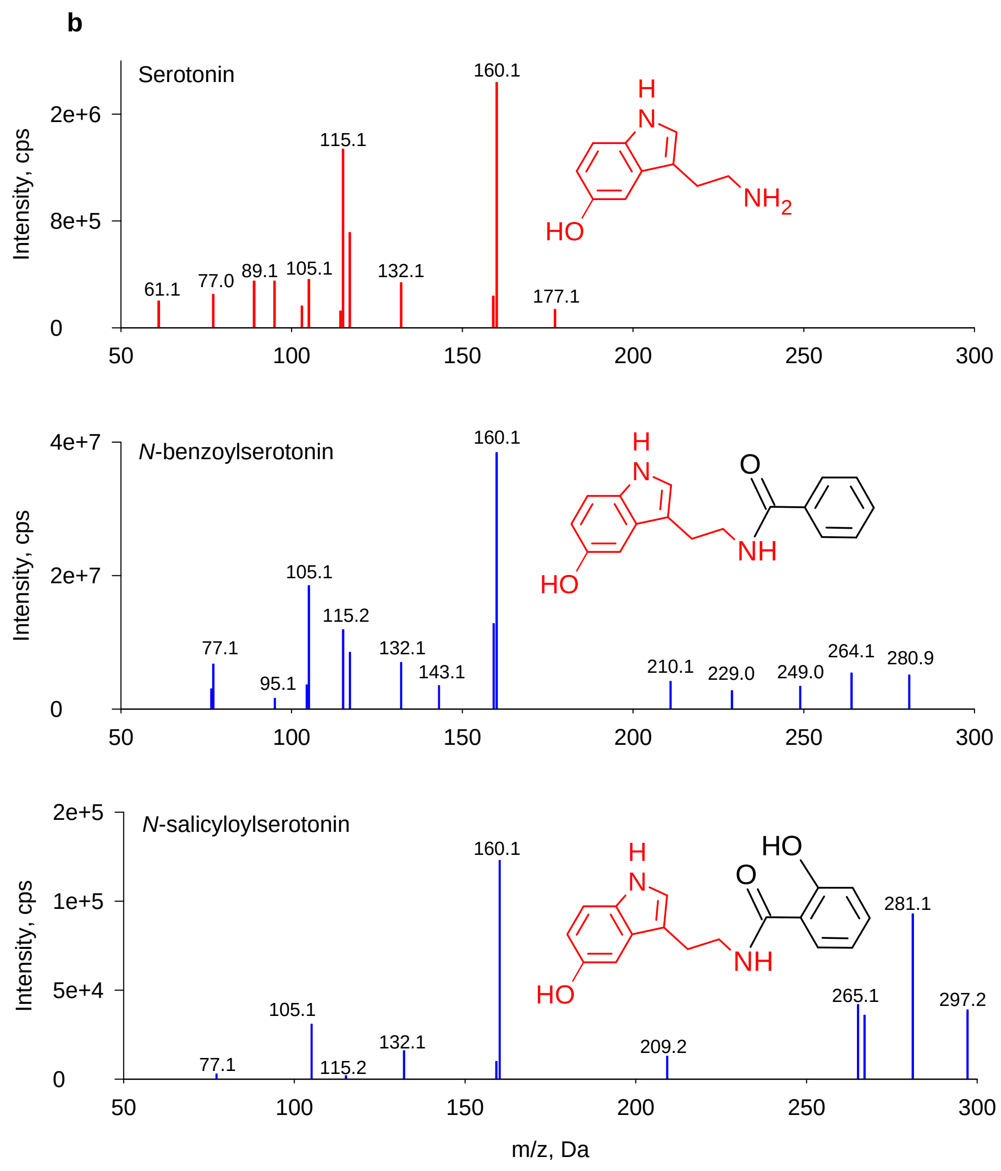

Supplementary Figure 8 The mass spectrum and structure of some metabolites for GGM results.

(**a**) Tryptamine related metabolites and (**b**) serotonin related metabolites.

mr896 (N-Benzoyltryptamine)

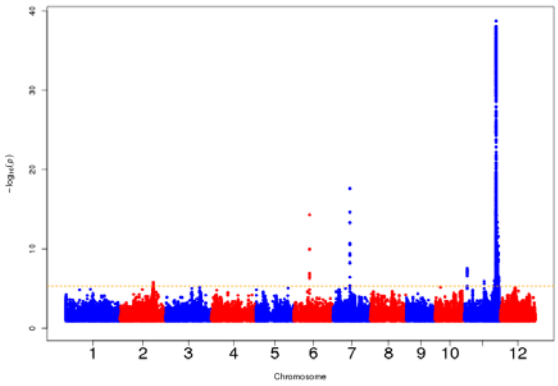

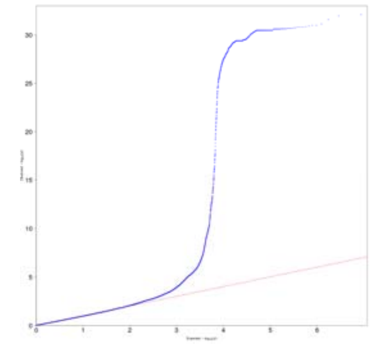

mr903 (N-Benzoylserotonin)

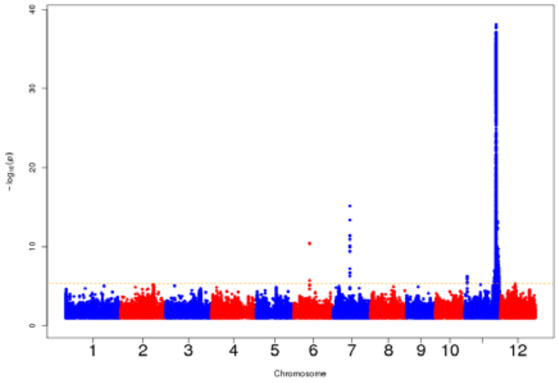

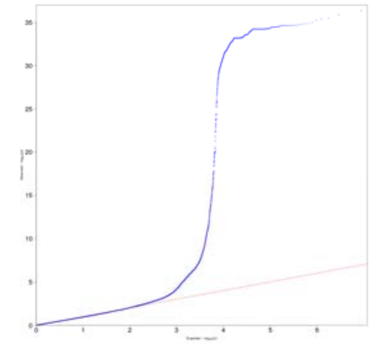

mr904 (N-Cinnamoyltryptamine)

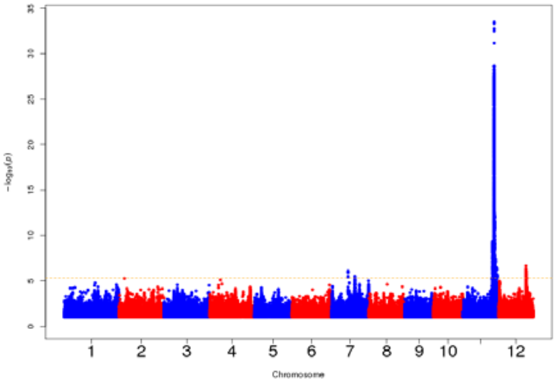

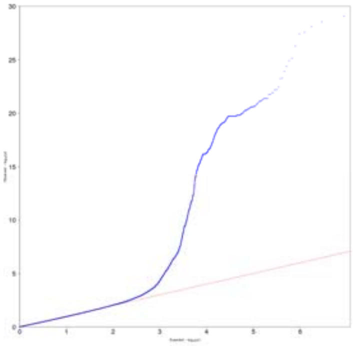

mr908 (N-Salicyloylserotonin)

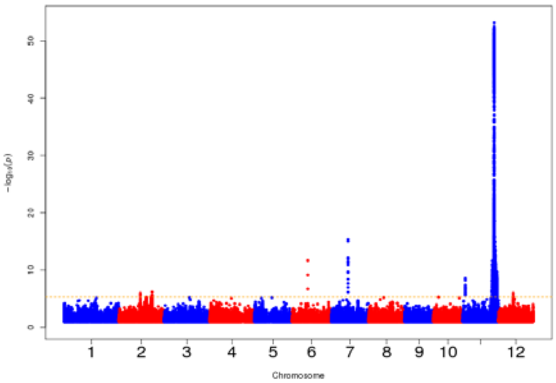

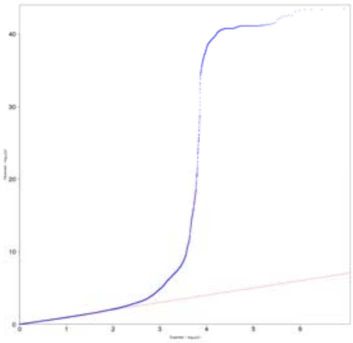

1000 grain weight

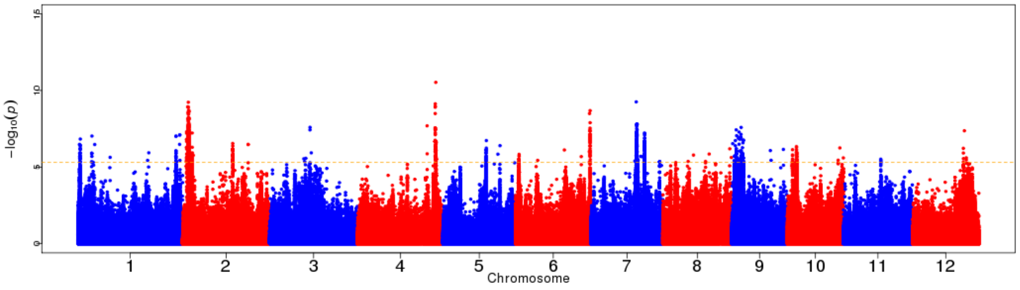

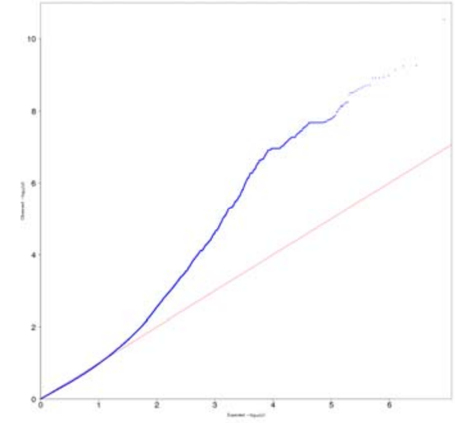

Grain length

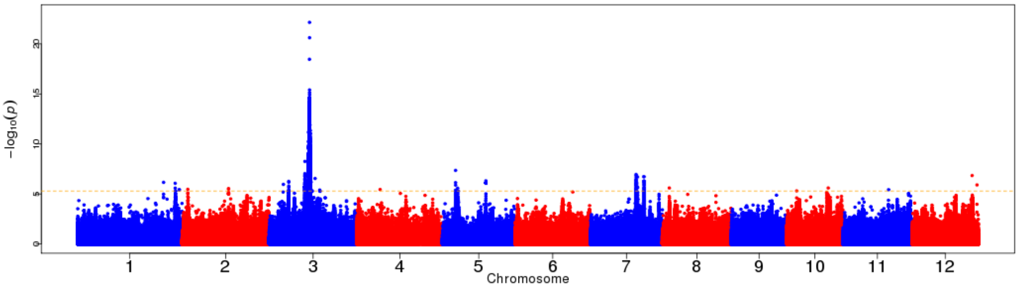

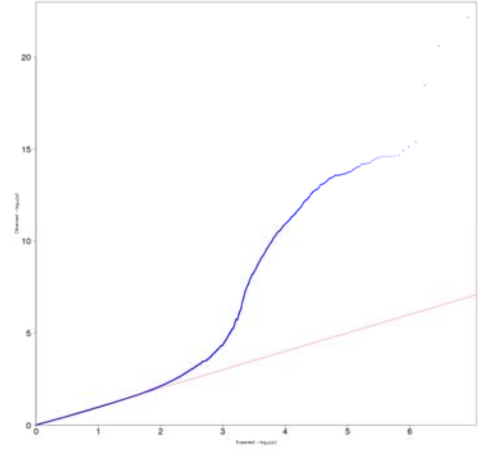

Grain thickness

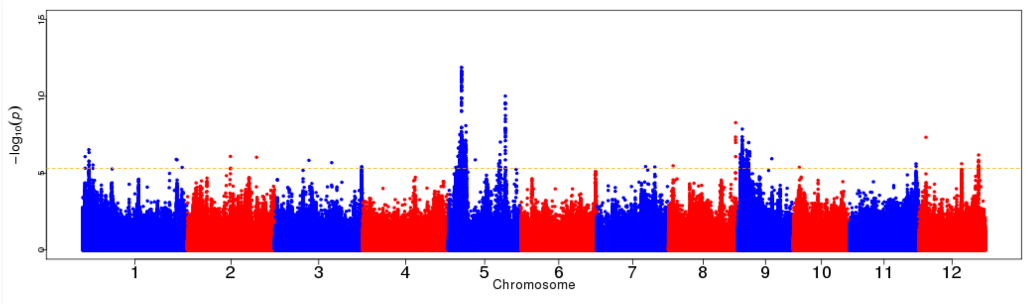

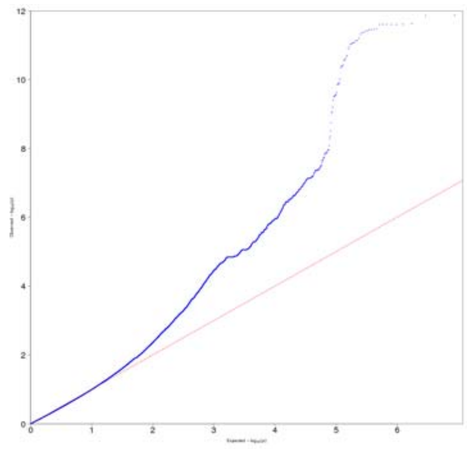

Grain width

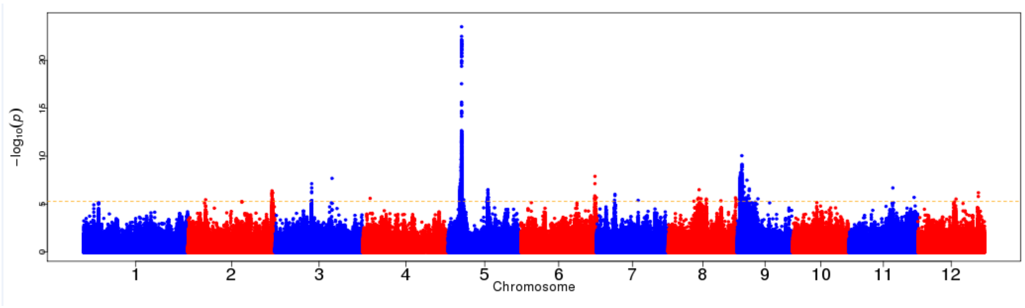

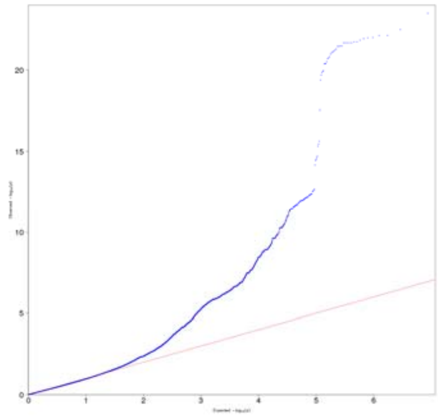

Hull color

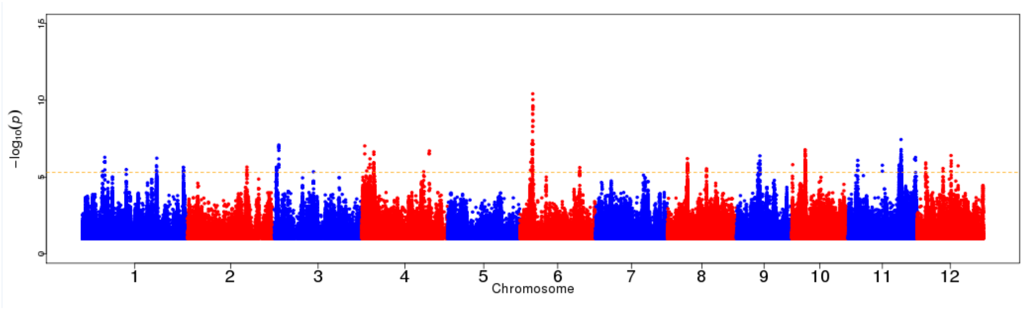

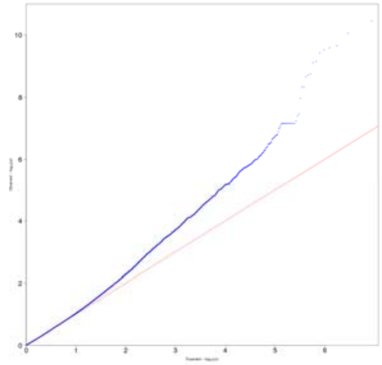

Seed color

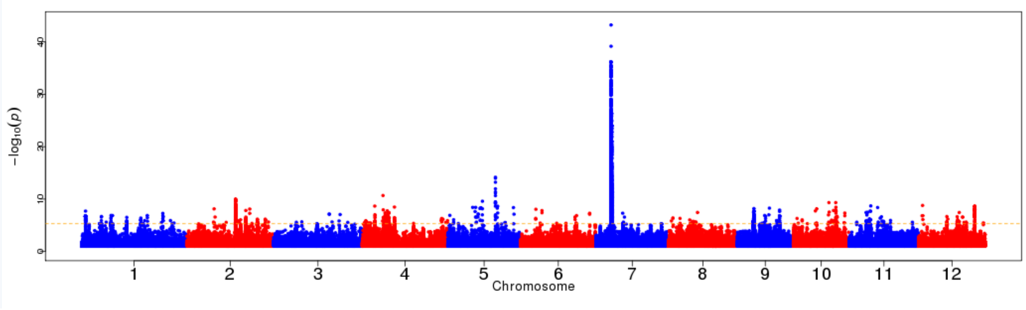

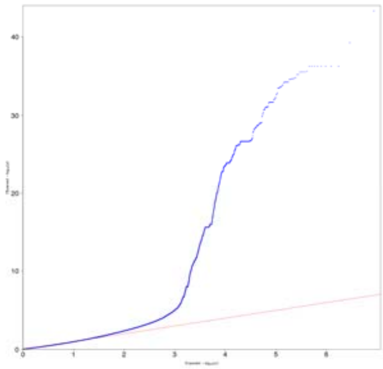

Supplementary Figure 9 The related Manhattan plots (up) and quantile-quantile plots (down).

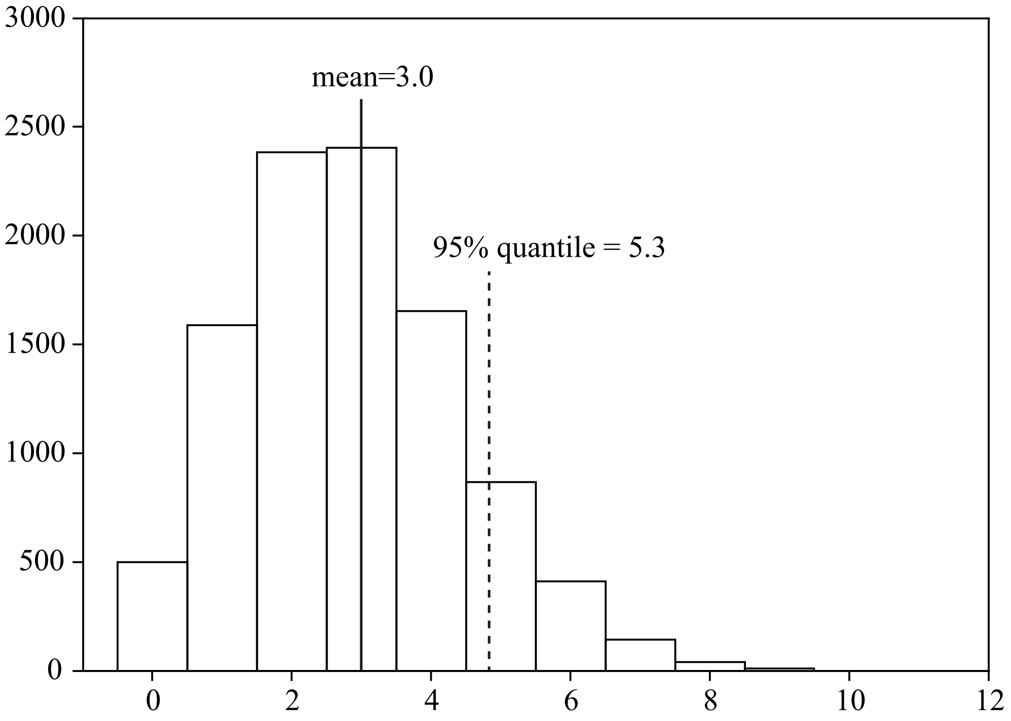

Supplementary Figure 10 Permutations of number of homologous or co-linear loci occurred by chance.

Given the number of loci studied between two species, an average of 3.0 out of 42 homologous or co-linear loci could possibly be due to chance alone. The 95% quantile of the distribution for metabolite- metabolite loci of homolog or co-linear is 5.3.

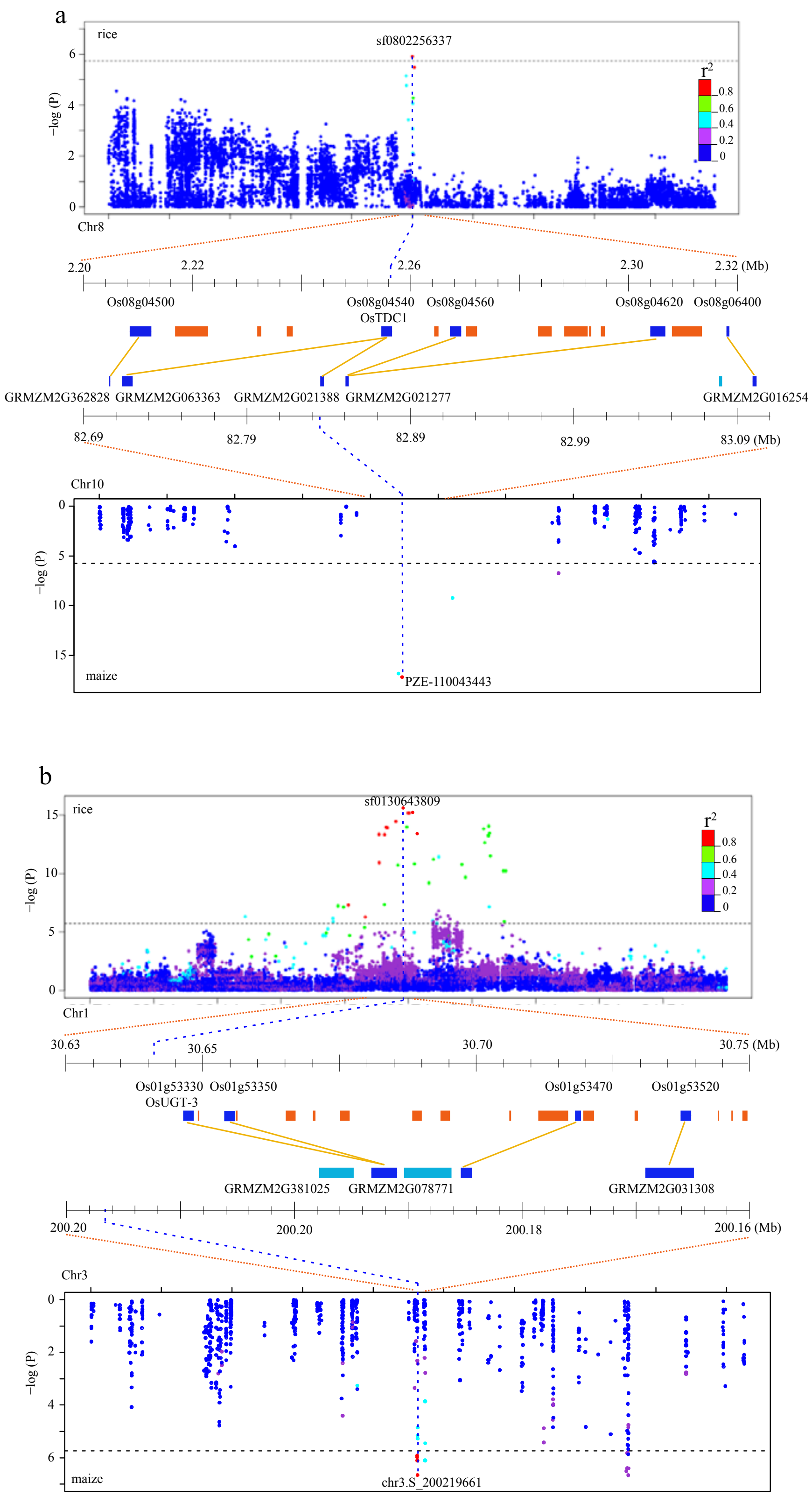

a
rice
sf0802256337
−log (P)
r2
0.8
0.6
0.4
0.2
0
Chr8
2.20
2.22
2.26
2.30
2.32 (Mb)
Os08g04500
Os08g04540
OsTDC1
Os08g04560
Os08g04620
Os08g06400
GRMZM2G362828
GRMZM2G063363
GRMZM2G021388
GRMZM2G021277
GRMZM2G016254
82.69
82.79
82.89
82.99
83.09 (Mb)
Chr10
maize
PZE-110043443
b
rice
sf0130643809
Chr1
30.63
30.65
30.70
30.75 (Mb)
Os01g53330
OsUGT-3
Os01g53350
Os01g53470
Os01g53520
GRMZM2G381025
GRMZM2G078771
GRMZM2G031308
200.20
200.20
200.18
200.16 (Mb)
Chr3
maize
chr3.S_200219661

c

140 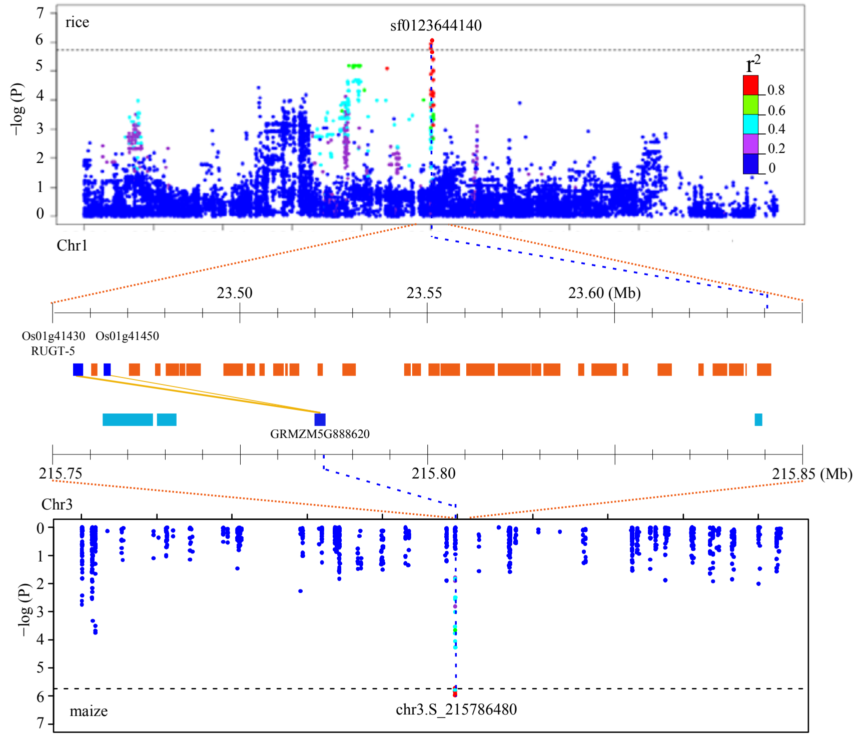
rice
sf0123644140
−log (P)
r2
0.8
0.6
0.4
0.2
0
Chr1
23.50
23.55
23.60 (Mb)
Os01g41430
RUGT-5
Os01g41450
GRMZM5G888620
215.75
215.80
215.85 (Mb)
Chr3
maize
chr3.S_215786480

d

141 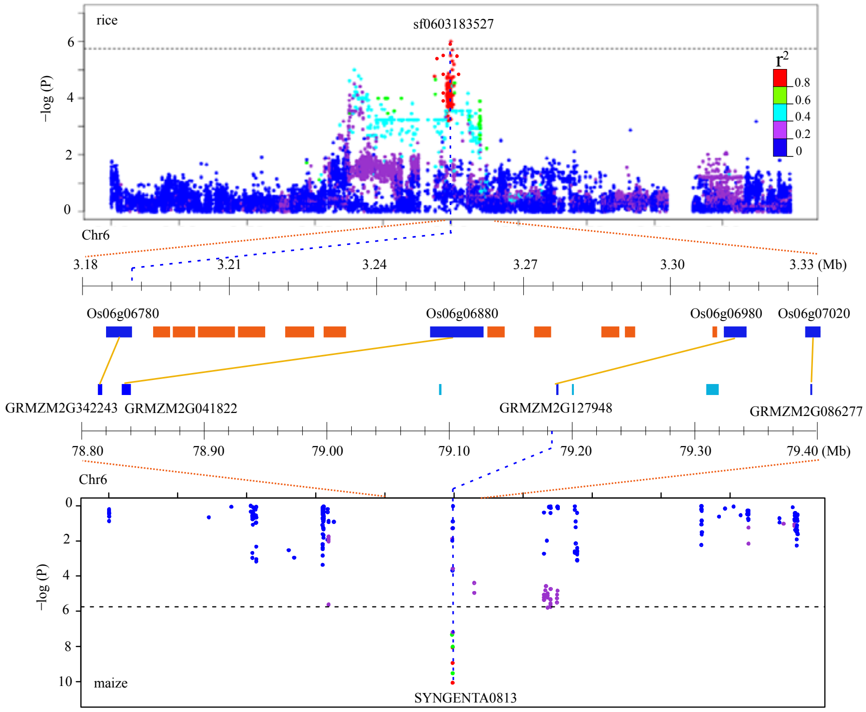
rice
sf0603183527
−log (P)
r2
0.8
0.6
0.4
0.2
0
Chr6
3.18
3.21
3.24
3.27
3.30
3.33 (Mb)
Os06g06780
Os06g06880
Os06g06980
Os06g07020
GRMZM2G342243
GRMZM2G041822
GRMZM2G127948
GRMZM2G086277
78.80
78.90
79.00
79.10
79.20
79.30
79.40 (Mb)
Chr6
maize
SYNGENTA0813

e

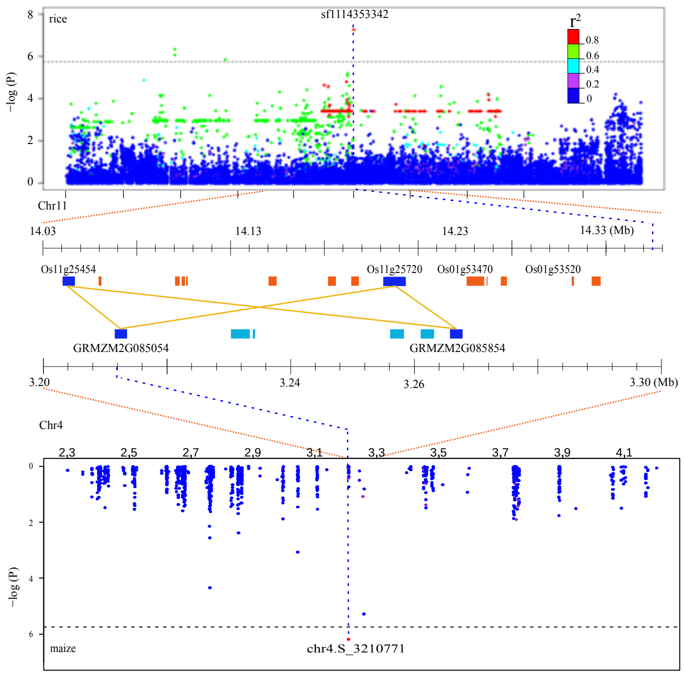

f

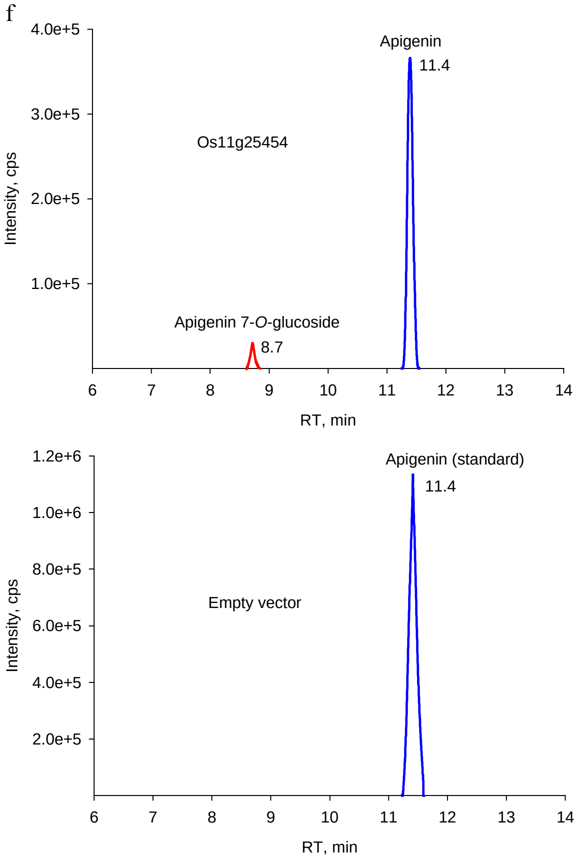

Supplementary Figure 11 Colinear genomic regions and the

homologous loci (or genes) of tryptamine (**a**), 3’, 4’, 5’-tricetin *O*-hexoside (**b**) chrysoeriol (**c**), caffeic acid (**d**) and apigenin 7-*O*-glucoside (**e**) between rice grain and maize kernel. (**f**) Annotation of *Os11g25454* as the candidate gene underlying the mGWAS for apigenin 7-*O*-glucoside and identified the function by *in vitro*. LC-MS chromatograms of *in vitro* enzyme assay shows the enzyme activity of recombinant Os11g25454 (up). Protein extract from *E. coli* containing pDEST15 empty vector were used as a negative control (down).

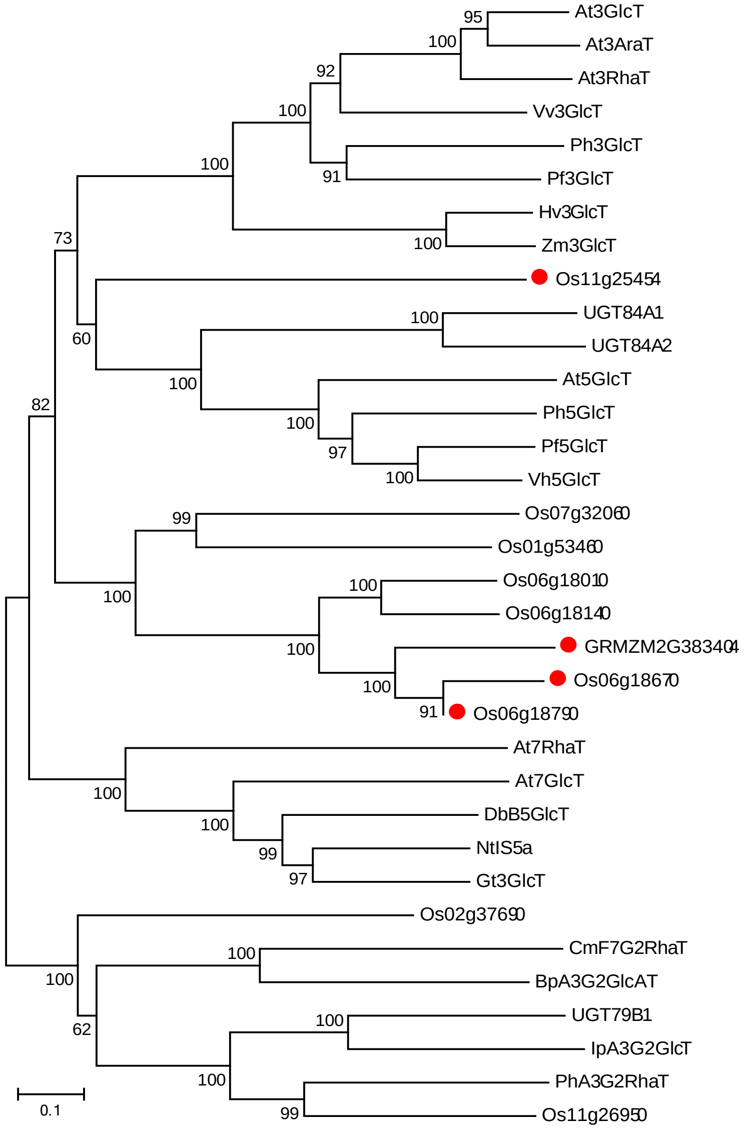

Supplementary Figure 12 Phylogenetic analysis of glucosyltransferase genes from the plant glucosyltransferase family.

The neighbor-joining tree was constructed using aligned full-length amino acid sequences. Bootstrap values from 1, 000 replicates are indicate at each node. Bar = 0.1 amino acid substitutions per site.

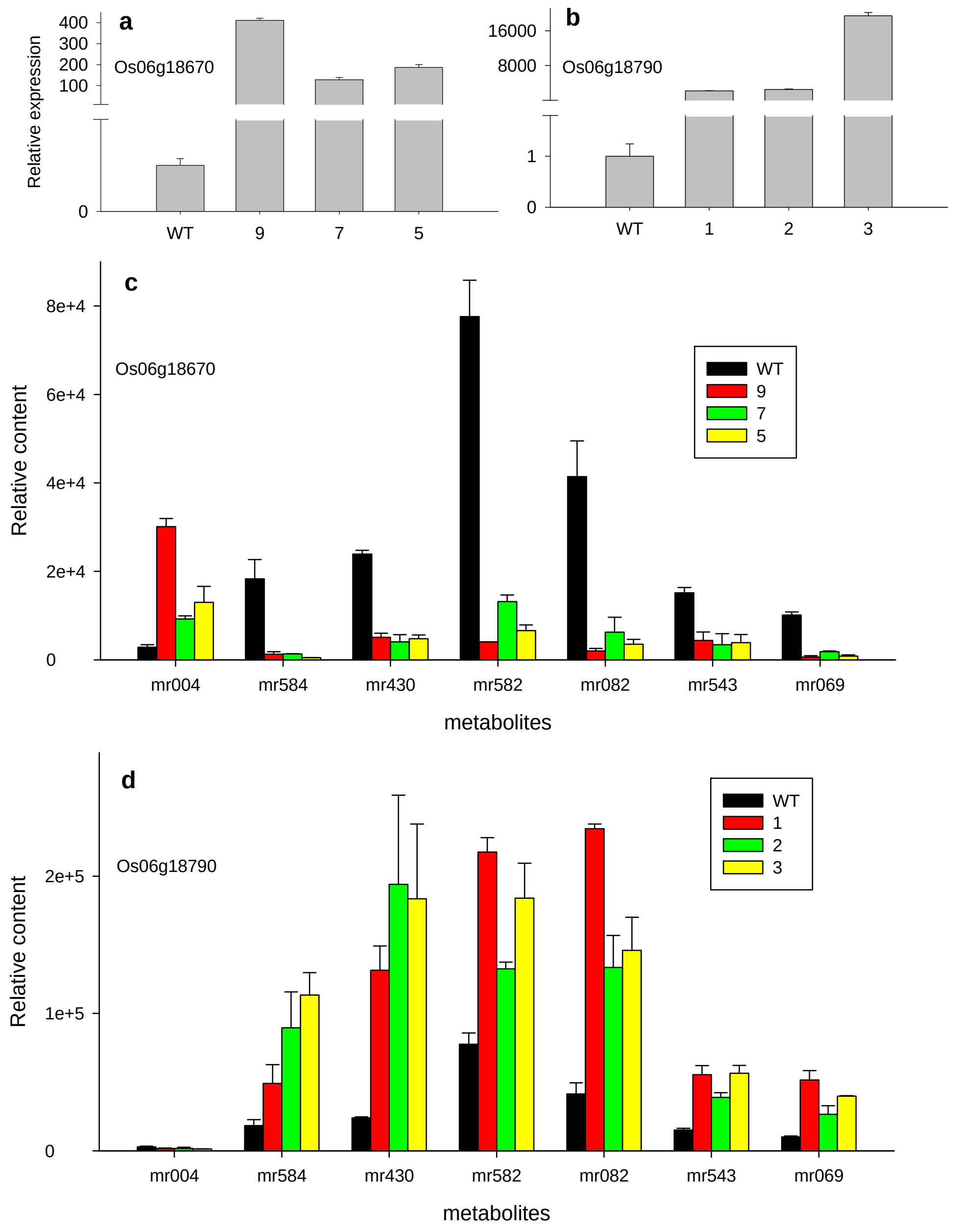

Supplementary Figure 13 The transgenic results of *Os06g18670* and *Os06g18790*.

The expression level of *Os06g18670* (**a**) and *Os06g18790* (**b**) respectively; (**c** and **d**), the relative content of some flavonoids in rice transgenic individuals. WT, the transgenic background variety ZH11. The

P value is calculated using the Student's *t* tests. Data are shown as the means ± s.e.m., n = 3. mr004, di-*C*,*C*-pentosyl-apigenin; mr584, *C*-hexosyl-luteolin *O*-*p*-coumaroylhexoside; mr430, Luteolin 6-*C*-glucoside; mr582, *C*-hexosyl-apigenin *O*-*p*-coumaroylhexoside; mr082, *C*-hexosyl-apigenin *O*-feruloylhexoside; mr543, *C*-hexosyl-chrysoeriol *O*-hexoside; mr069, di-*C*, *C*-hexosyl-apigenin derivative.

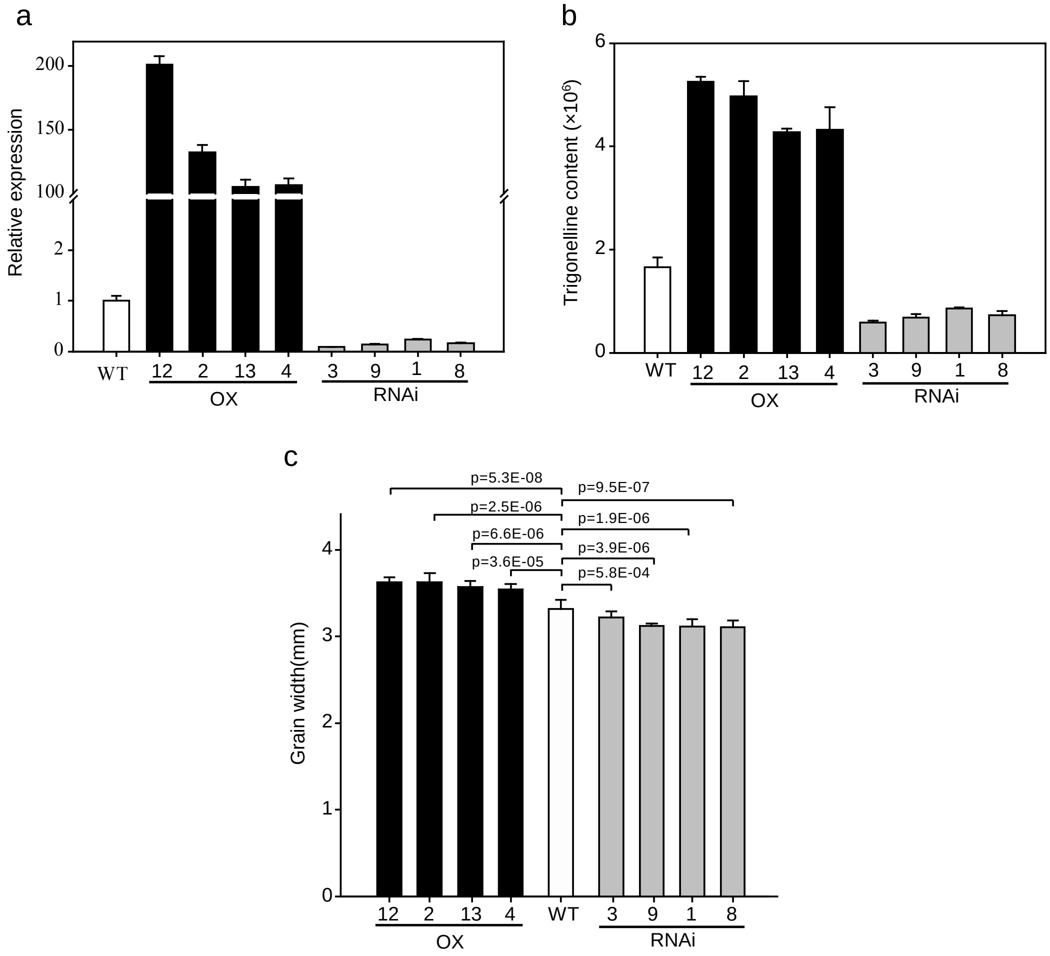

Supplementary Figure 14. Phenotype data in transgenic plants.

Shown are bar plots for the mRNA level of *Os02g57760* (**a**) and for the content of trigonelline (**b**) in transgenic positive individuals. (**c**) The comparison of grain width between transgenic plants and wild type. The *P* value is calculated using the Student's *t* tests. Data are shown as the means ± s.e.m., n = 3.

Supplementary Note 1

Metabolites identification and putative annotation strategies.

We determined the relative levels of 837 distinct metabolic traits in rice grains using a newly developed liquid chromatography-tandem mass spectrometry (LC-MS/MS)-based, widely targeted metabolic profiling method[1]. Of the 837 metabolic features, 80 were identified based on comparisons of MS/MS spectra, an exact mass number, and retention time with those of authentic standards (**Supplementary Data 2**). A total of 230 were putatively annotated based on high resolution MS, MS/MS spectra and other strategies, including: i) linking the unknown metabolites to functionally related genes based on genetic mapping[2], and/or further verifying the genes using *in vivo* or *in vitro* strategies; ii) connecting two metabolites with similar MS/MS spectra[3]; and iii) combining the Gaussian graphical model (GGM)[4] with the similarity of the MS/MS spectra. The details are provided below.

To annotate more metabolites, we associated the unknown metabolites with annotated genes based on our high-resolution genetic mapping as previously reported[2], For example, the strong association between SNP sf0233224406 located 22 kb from *OsLKR*[5] (encoding saccharopine dehydrogenase) and the mr208 (m/z 277) level suggested that this metabolite could be saccharopine or its derivative. We subsequently identified the mr208 metabolite as saccharopine by comparing the retention time and fragmentation pattern of this metabolite with the commercial standard (**Supplementary Figure 1**).

The strong association between SNP sf0605268699 located 45 kb from *OsC1* (encoding MYB transcription factor)[6] and the mr063 (m/z 465.1033) level suggested that this metabolite could be an anthocyanin or its derivative. We subsequently identified the mr063 metabolite as delphinidin 3-*O*-glucoside by high resolution MS and the fragmentation pattern of this metabolite (**Supplementary Figure 2**). Using this approach, the mGWAS enabled the putative annotation of more than 40 metabolites (**Supplementary Data 4**).

We used the Gaussian graphical model (GGM) to reconstruct pathways involving directly related metabolites. GGM is based on pairwise Pearson correlation coefficients conditioned against the correlation with all other metabolites[4]. First, we performed Principal component analysis (PCA) on the genotype mean values to summarize the correlations and pinpoint groups of correlated metabolites. We found some obvious clusters, such as the class of flavonoids, some amino acids and terpenoids etc., suggesting strong correlations between them (**Supplementary Fig. 7a**). For the GGM calculation in this article, a full data matrix was constructed from 502 samples and 587 metabolites. GGM with an empirical Bayes approach[7] was employed to estimate partial correlations and reconstruct a GGM network from a given dataset. In addition to the results for the full population, we included the data for separate GGM analyses across the 2 genetic subgroups (*indica* and *japonica*). We compared the GGM

networks of three groups filtered by a significant *P*-value < 2.9E-07 based on the Bonferroni correction. Together, the resulting GGM consists of a total of 2119 connections (**Supplementary Data 15**). In accordance with previous observations, we consistently observed associations between biochemically related metabolites from various metabolic pathways in both the overall network (**Supplementary Fig. 7b**) and the top list of high-scoring GGM edges: metabolites naringenin 7-*O*-glucoside and apigenin 7-*O*-glucoside (*P*-cor = 0.30, Pearson's correlation coefficient), which are involved in flavonoid metabolism, or threonyl carbamoyl adenosine and [1,2,4]triazolo[1,5-a]pyrimidine-7-carboxamide,4,5,6,7-tetrahydro-N-(2-methoxy-5-methylphenyl)-5-oxo- (*P*-cor = -0.08, Pearson's correlation coefficient), which represent related nucleic acid derivatives (**Supplementary Fig. 7b**). Then, we searched for high-score correlating pairs of an unknown and a known metabolite that might provide a biochemical context for the unknown metabolite. For example, the correlation between tryptamine (mr653) and mr904 was 0.09. This pair had the same major m/z 144 fragment (the main ion for tryptamine), suggesting that mr904 was a tryptamine derivative. We putatively annotated mr904 as *N*-cinnamoyltryptamine by comparing the MS and fragmentation patterns with tryptamine (**Supplementary Data 15**). Over 30 metabolites were putatively annotated using this approach

(**Supplementary Data 4** and **15**).

Supplementary Note 2

The process and criterion for the assignment of candidate genes responsible for the variation of metabolic traits based on mGWAS.

To confirm the candidate genes responsible for the variation of metabolic traits, we mined the candidate genes using the following methods: i) estimating the allelic effect of each genotypic class in close proximity to the most significant peak SNPs and confirming the associated SNP/InDel; ii) looking for a protein or protein cluster that was biochemically and/or biologically related to the associated metabolic trait encoded at these loci; iii) performing cluster analysis of the candidate genes relative to homologous genes with known functions; iv) cross-referencing with results from linkage mapping and v) verifying the candidate genes according to the tissue-specific expression pattern.

For example, SNP sf0310132518 located 12 kb from *Os03g18130* (encoding a putative asparagine synthetase) was significantly associated (P = 5.7E-07, LMM, n = 502) with asparagine (mr173). The high sequence identity (68% at the amino acid level) between *Os03g18130* and *AtASN2* suggested that *Os03g18130* encoded an asparagine synthetase. This hypothesis was supported by the preferential expression of *Os03g18130* together with a higher accumulation of this metabolite in the

rice grain.

We also observed a 2 bp deletion that resulted in a frame shift in *Os04g11970*. This deletion was highly significantly associated with the variation (*P* = 6.7E-47, LMM, n = 502) and the absence of *O*-methylapigenin *C*-hexoside, strongly suggesting the loss of function allele for this candidate.

SNP sf0524319598 located in *Os05g41645* (encoding a putative chalcone synthase), was significantly associated (*P* = 3.3E-52, LMM, n = 502) with *C*-pentosyl-apigenin *O*-rutinoside (mr080). The high sequence identity (48% at the amino acid level) between *Os05g41645* and *AtTT4* suggested that *Os05g41645* encoded a chalcone synthase underlying this flavonoid.

SNP sf0137818225 located 27 kb from *Os01g65260* (encoding a putative amido phosphor ribosyltransferase) was significantly associated (*P* = 2.5E-10, LMM, n = 502) with threonyl carbamoyl adenosine (mr408). The above data strongly suggested that *Os01g65260* encoded an amido phosphor ribosyltransferase that was involved in the accumulation of threonyl carbamoyl adenosine.

Together, more than 30 candidate genes were newly disclosed by examining the mGWAS data from the rice grain alone in addition to 30 genes that were previously identified in studies using either mutants or recombinant and natural populations (**Supplementary Data 14**).

Supplementary Note 3

Comparative mGWAS between rice and maize.

Comparative linkage mapping between crop plants, such as wheat, maize, and rice[8,9], has revealed good correspondences among QTLs in crop plants for traits including seed size, shattering habit, and flowering time etc., and has been suggested as a useful tool for predictions of the loci of homologous major genes[10-12]. This concept was modified and extended in our mGWAS for candidate gene mining based on the co-linear mapping of the targeted metabolic trait(s) between species (e.g., searching for candidates within homologous or co-linear regions co-mapped by the same metabolites detected in both species). Because orthologous genes between rice and maize may vary in their substrate specificity (e.g., responsible for similar but not exactly the same metabolite), metabolites with similar structures were also included in the comparison.

In this study, rice (Nipponbare, MSU version 6.1) and maize (B73, RefGen_v2) genomes were used for the identification and characterization of homologous regions. The sequence alignment analysis was based on a VISTA sequence alignment algorithm program[13]. Detailed information concerning the homologous fragments between the two species is available from the VISTA database (http://genome.lbl.gov/vista/index.shtml)[14].

We previously performed metabolic profiling of 983 metabolic features

in 702 diverse maize accessions and identified hundreds of significant locus-trait associations in maize kernel through mGWAS[15]. To investigate the common genetic control of metabolism between rice and maize, we focused on the 123 co-detected metabolic features in rice grains and maize kernels (**Supplementary Data 4**). The co-detected metabolic traits in both species were used to filter out loci through mGWAS in the rice and maize grain. The calculated genome-wide threshold was set at P = 1.8E-06 (MLM, n = 339) for maize[17] and P = 1.3E-06, 1.8E-06 and 4.1E-06 (LMM, n = 502) for the whole panel, *indica* and *japonica* rice[2], respectively. According to these thresholds, we obtained a total of 420 (**Supplementary Data 16**) and 292 (**Supplementary Data 17**) loci for the 123 co-detected metabolic features in rice and maize, respectively. We searched for homologous loci mapped by the same metabolites or metabolites of similar structures between 2 species by referring to the VISTA database (http://genome.lbl.gov/vista/index.shtml) and detected 42 loci for 23 metabolites or metabolites of similar structures in both species (**Supplementary Data 18**).

To test the significance of our GWAS homolog or co-linearity, we adopted the randomization test of Churchill et al. 1994[16] to determine the proportion of overlaps expected to occur by chance. The deviation from the random number of GWAS homolog or co-linearity was calculated as

follows. All SNP hits of co-detected metabolites were randomly distributed over the 420 and 292 identified association positions in the rice and maize kernel, respectively. Then, we counted the number of homolog or co-linearity with each locus for metabolites with the same or similar structures using rice and maize fragments according to their local LD decays. This procedure was repeated 10,000 times and yielded a distribution of expected numbers of loci of homolog or co-linearity. Then, this distribution was compared against the outcome for the actual data. The mean and 95% quantile of the distribution for tge metabolite-metabolite loci of homolog or co-linearity were 3.0 and 5.3, respectively (**Supplementary Fig. 10**), suggesting that the majority of the observed overlaps could not possibly be explained by chance alone.

Next, we looked for homologous gene(s) within the homologous or co-linear loci between rice and maize using an expectation value (E) of 10^{-10} as the significance threshold[17]. Using this approach, a number of candidate genes were assigned (**Supplementary Fig. 11** and **Supplementary Data 19**), including reported genes for metabolic traits such as tryptophan decarboxylase *OsTDC1* (*Os08g04540*), which catalyzes the conversion of tryptophan into tryptamine in rice[18] (**Supplementary Fig. 11a**), and another two flavonoid *O*-UDP-glucosyl transferases (*OsUGT-3*[19] and *RUGT-5*[20]) underlying the variation of 3’, 4’, 5’-tricetin *O*-hexoside and chrysoeriol, respectively (**Supplementary**

Figs. 11b-c).

Supplementary References

1. Chen, W. *et al.* A novel integrated method for large-scale detection, identification, and quantification of widely targeted metabolites: application in the study of rice metabolomics. *Mol Plant* **6,** 1769-1780 (2013).
2. Chen, W. *et al.* Genome-wide association analyses provide genetic and biochemical insights into natural variation in rice metabolism. *Nat Genet* **46,** 714-721 (2014).
3. Matsuda, F. *et al.* Metabolome-genome-wide association study dissects genetic architecture for generating natural variation in rice secondary metabolism. *Plant J* **81,** 13-23 (2015).
4. Krumsiek, J. *et al.* Mining the unknown: a systems approach to metabolite identification combining genetic and metabolic information. *PLoS Genet* **8,** e1003005 (2012).
5. Kawakatsu, T. & Takaiwa, F. Differences in transcriptional regulatory mechanisms functioning for free lysine content and seed storage protein accumulation in rice grain. *Plant Cell Physiol* **51,** 1964-1974 (2010).
6. Saitoh, K., Onishi, K., Mikami, I., Thidar, K. & Sano, Y. Allelic diversification at the C (OsC1) locus of wild and cultivated rice: nucleotide changes associated with phenotypes. *Genetics* **168,** 997-1007 (2004).
7. Schafer, J. & Strimmer, K. An empirical Bayes approach to inferring large-scale gene association networks. *Bioinformatics* **21,** 754-764 (2005).
8. Moore, G., Devos, K.M., Wang, Z. & Gale, M.D. Cereal genome evolution. Grasses, line up and form a circle. *Curr Biol* **5,** 737-739 (1995).
9. Ahn, S. & Tanksley, S.D. Comparative linkage maps of the rice and maize genomes. *Proc Natl Acad Sci U S A* **90,** 7980-7984 (1993).
10. Lin, Y.R., Schertz, K.F. & Paterson, A.H. Comparative analysis of QTLs affecting plant height and maturity across the Poaceae, in reference to an interspecific sorghum population. *Genetics* **141,** 391-411 (1995).
11. Ming, R. *et al.* Comparative analysis of QTLs affecting plant height and flowering among closely-related diploid and polyploid genomes. *Genome* **45,** 794-803 (2002).
12. Paterson, A.H. *et al.* Convergent domestication of cereal crops by independent mutations at corresponding genetic Loci. *Science* **269,** 1714-1718 (1995).
13. Frazer, K.A., Pachter, L., Poliakov, A., Rubin, E.M. & Dubchak, I. VISTA: computational tools for comparative genomics. *Nucleic Acids Res* **32,** W273-279 (2004).
14. Mayor, C. *et al.* VISTA : visualizing global DNA sequence alignments of arbitrary length. *Bioinformatics* **16,** 1046-1047 (2000).
15. Wen, W. *et al.* Metabolome-based genome-wide association study of maize kernel leads to novel biochemical insights. *Nat Commun* **5,** 3438 (2014).
16. Churchill, G.A. & Doerge, R.W. Empirical threshold values for quantitative trait mapping. *Genetics* **138,** 963-971 (1994).
17. Dean, R.A. *et al.* The genome sequence of the rice blast fungus Magnaporthe grisea. *Nature* **434,** 980-986 (2005).
18. Kang, S., Kang, K., Lee, K. & Back, K. Characterization of rice tryptophan decarboxylases and

their direct involvement in serotonin biosynthesis in transgenic rice. *Planta* **227,** 263-272 (2007).

19. Kim, B.G. *et al*. Flavonoid *O*-diglucosyltransferase from rice: molecular cloning and characterization. *J Plant Biol* **52,** 41-48 (2009).
20. Ko, J.H., Kim, B.G., Hur, H.G., Lim, Y. & Ahn, J.H. Molecular cloning, expression and characterization of a glycosyltransferase from rice. *Plant Cell Rep* **25,** 741-746 (2006).
